# Description and genome analysis of a novel archaeon isolated from a syntrophic pyrite-forming enrichment culture and reclassification of *Methanospirillum hungatei* strains GP1 and SK as *Methanospirillum purgamenti* sp. nov.

**DOI:** 10.1371/journal.pone.0308405

**Published:** 2024-08-26

**Authors:** Nathalie Pradel, Manon Bartoli, Michel Koenen, Nicole Bale, Meina Neumann-Schaal, Cathrin Spröer, Boyke Bunk, Manfred Rohde, Michael Pester, Stefan Spring

**Affiliations:** 1 CNRS/INSU, IRD, MIO, UM 110, Aix-Marseille Université, Université du Sud Toulon-Var, Marseille, France; 2 Royal Netherlands Institute for Sea Research, Texel, Netherlands; 3 Research Group Metabolomics, Leibniz Institute DSMZ-German Collection of Microorganisms and Cell Cultures, Braunschweig, Germany; 4 Department Bioinformatics, Leibniz Institute DSMZ–German Collection of Microorganisms and Cell Cultures, Braunschweig, Germany; 5 Central Facility for Microscopy, Helmholtz Centre for Infection Research, HZI, Braunschweig, Germany; 6 Department Microorganisms, Leibniz Institute DSMZ-German Collection of Microorganisms and Cell Cultures, Braunschweig, Germany; 7 Institute for Microbiology, Technical University of Braunschweig, Braunschweig, Germany; The University of Akron, UNITED STATES OF AMERICA

## Abstract

The archaeal isolate J.3.6.1-F.2.7.3^T^ was obtained from an anaerobic enrichment culture, where it may play an important role in methane production during pyrite formation. The new isolate formed a species-level clade with *Methanospirillum hungatei* strains GP1 and SK, which is separate from the type strain JF-1^T^. Cultivation-independent surveys indicate the occurrence of this phylogenetic group in sediments and anaerobic digesters. The abundance of this clade appears to be negatively affected by high nitrogen loads, indicating a sensitivity to certain nitrogen compounds that is not known in *M*. *hungatei* JF-1^T^. The relatively large core genome of this *Methanospirillum* clade is indicative of niche specialization and efficient control of horizontal gene transfer. Genes for nitrogenase and F_420_-dependent secondary alcohol dehydrogenase contribute to the metabolic versatility of this lineage. Characteristics of the new isolate such as the ability to utilize 2-propanol as an electron donor or the requirement for acetate as a carbon source are found also in the strains GP1 and SK, but not in the type strain *M*. *hungatei* JF-1^T^. Based on the genomic differences to related species, a new species within the genus *Methanospirillum* is proposed with the name *M*. *purgamenti* sp. nov. The determined phenotypic characteristics support this proposal and indicate a metabolic adaptation to a separate ecological niche.

## Introduction

The metabolic activity and composition of an enrichment culture producing pyrite and methane from FeS, H_2_S, and CO_2_ under anaerobic and mesophilic conditions have recently been described in detail [1]. It has been found that the transformation of iron monosulfide to pyrite is coupled with methanogenesis.

In addition, cultivation-independent population analyses have shown that methanogens make up a significant proportion of the Archaea in these cultures. Based on the retrieved 16S rRNA gene sequences a close relationship to a *Methanospirillum* clade has been deduced, which is frequently found in engineered anoxic environments like bioreactors. The best-studied representative of this group is the species *M*. *hungatei*, which is known for its growth under H_2_-limiting conditions [2] and its syntrophic interaction with fatty acid-degrading bacteria [3, 4].

It is thought that syntrophy plays also an important role in the microbially mediated production of pyrite under mesophilic and anoxic conditions. Two reaction mechanisms are currently discussed here, which both would depend on a hydrogen scavenging reaction, *e*.*g*. by a hydrogenotrophic methanogen. In the first one, pyrite would be formed directly from hydrogen sulfide and iron monosulfide under the release of molecular hydrogen. In the second one, the transformation of iron monosulfide to pyrite would involve the oxidation of sulfide to zerovalent sulfur, which is energetically unfavorable in the absence of a suitable electron acceptor [1, 5].

Interestingly, it has recently been shown that the oxidation of sulfide could be coupled to methanogenesis by interspecies electron transfer between sulfide-oxidizing bacteria and electrotrophic methanogens, so that the reaction can proceed in the direction of sulfur [6]. In the reported experimental setup, the syntrophic coupling of sulfide oxidation with methanogenesis was dependent on electrically conductive magnetite particles, which was not detected in the pyrite-forming enrichment cultures. However, it has been shown that the *Methanospirillum* species *M*. *hungatei* forms electrically conductive archaella [7] that could play a role in the long-range electron transfer between cells of different species.

In the present study, a novel *Methanospirillum* isolate was obtained representing a phylotype previously obtained by PCR-based sequencing of 16S rRNA genes in a pyrite-forming enrichment culture [1]. The new isolate was further characterized and compared with similar strains from different environments. The results of this study, together with the knowledge previously gained from isolates of these enrichments with the ability to reduce sulfur [8] or dismutate oxidized sulfur compounds [9], will contribute to the design of experimental setups that could lead to a better understanding of the role of methanogens and associated microorganisms in pyrite formation.

## Materials and methods

### Isolation of strain J.3.6.1-F.2.7.3^T^ and cultivation of pure cultures

The source for the isolation of methanogenic archaea was the pyrite-forming mixed culture J3, which is a parallel culture of J5 as described in detail previously [1]. The medium used for the enrichment of methanogens from the mixed culture was based on DSMZ medium 119 [10], but complex organic nutrients were omitted to inhibit the growth of heterotrophic bacteria. It was designated as medium I and has the following composition (per liter of distilled water): 0.50 g KH_2_PO_4_, 0.40 g NH_4_Cl, 0.40 g NaCl, 0.40 g MgCl_2_ × 6 H_2_O, 0.05 g CaCl_2_ × 2 H_2_O, 1.00 g sodium acetate, 2.00 g sodium formate, 1.00 g Na_2_CO_3_, 20.00 ml fatty acid mixture (from DSMZ medium 119), 1.00 ml trace elements solution (from DSMZ medium 320), 1.00 ml selenite-tungstate solution (from DSMZ medium 385), 1.00 ml Wolin’s vitamin solution (from DSMZ medium 120), 0.50 ml sodium resazurin solution (0.1% w/v), 0.50 g L-cysteine-HCl × H_2_O, 0.50 g Na_2_S × 9 H_2_O. For routine cultivation, a medium II with the following composition was designed (per liter of distilled water): 0.20 g KH_2_PO_4_, 0.25 g NH_4_Cl, 1.00 g NaCl, 0.40 g MgSO_4_ × 7 H_2_O, 0.50 g KCl, 0.15 g CaCl_2_ × 2 H_2_O, 0.80 g sodium acetate, 1.00 g Na_2_CO_3_, 1.00 ml trace elements solution (from DSMZ medium 320), 1.00 ml selenite-tungstate solution (from DSMZ medium 385), 1.00 ml Wolin’s vitamin solution (from DSMZ medium 120), 0.50 ml sodium resazurin solution (0.1% w/v), 0.50 g Na_2_S × 9 H_2_O. This medium was included in the Media*Dive* database (https://mediadive.dsmz.de/) as DSMZ medium 1718 [10]. Cultivation media were prepared under 80% H_2_ and 20% CO_2_ gas atmosphere following the procedures described by Hungate and modified by Bryant [11]. The pH of the complete media was adjusted to 7.2. Cultures of the new isolate were incubated in the dark at 28°C.

For comparative studies the following strains were obtained from the open collection of the Leibniz-Institute DSMZ: *Methanospirillum hungatei* SK (DSM 3595), *M*. *hungatei* JF-1^T^ (DSM 864^T^), *M*. *lacunae* Ki8-1^T^ (DSM 22751^T^), and *M*. *stamsii* Pt1^T^ (DSM 26304^T^). The strains DSM 864^T^ and DSM 26304^T^ were cultured in the DSMZ medium 1718, strain DSM 3595 in DSMZ medium 374 and strain 22751^T^ in DSMZ medium 1273 [10]. Incubation temperatures were 30°C for DSM 22751^T^ and DSM 26304^T^, but 37°C for DSM 861^T^ and DSM 3595.

Chemicals and antibiotics used for the preparation of media were obtained in research grade from Merck KGaA (Darmstadt, Germany).

### Environmental distribution and reconstruction of phylogenetic relationships

The distribution of archaea in the environment representing the same species-level clade as the new isolate J.3.6.1-F.2.7.3^T^ was examined using a complete 16S rRNA gene sequence of this strain (CP075546:133354–134819) in a standard BLAST search [12] against a distinct NCBI nucleotide collection database, which is restricted to sequences from prokaryotes (Prokaryote nucleotide collection, nt_prok). Positive hits were assigned to the same taxonomic unit if they had at least 99% sequence identity with at least 90% query coverage. In addition, several wastewater metagenome datasets from the NCBI Sequence Read Archive (SRA) were queried using the 16S rRNA gene sequence of the new isolate to determine the presence of closely related strains in this environment.

Phylogenetic analyses were performed based on both alignments of individual genes and concatenated amino acid sequences of 122 conserved archaeal proteins [13]. Phylogenetic trees based on 16S rRNA genes were reconstructed using an alignment of almost complete 16S rRNA gene sequences contained in the SILVA database SSU Ref NR 99 release 138.1 [14]. Phylogenetic inferences were limited to a selection of 53 sequences, including all type strains of the order *Methanomicrobiales* and several environmental sequences representing metagenome-assembled genomes (MAGs) of uncultured archaea related to the newly isolated strain J.3.6.1-F.2.7.3^T^ (S1 Table). Maximum likelihood trees were reconstructed using RaxML (version 8.2.11) implemented in the ARB software package [15], with the rapid bootstrap analysis algorithm and the GTRGAMMA model for DNA evolution as settings.

Amino acid sequences of the methyl-coenzyme M reductase subunit alpha (McrA) were obtained from the NCBI Entrez Protein database or extracted from the available genome sequence following identification by a BLAST search using a reference sequence of McrA (S1 Table). If several isozymes of McrA occurred in a strain that were assigned to phylogenetically distant groups, the isozyme whose phylogenetic position correlated with the evolution of the 16S rRNA gene was retained as the possibility of misidentified McrA proteins cannot be excluded. An alignment of 61 McrA sequences was generated using the MUSCLE web interface from EMBL-EBI [16]. The resulting data set was submitted to the IQ-Tree web server version 1.6.12 [17] for the reconstruction of maximum likelihood trees using default settings. IQ-TREE software includes the ModelFinder program to select the most appropriate model for accurate phylogenetic estimates [18] and computes branch support using an ultrafast bootstrap approximation (UFBoot2) [19].

A total of 48 genomes, including most of the available type strains of *Methanomicrobiales*, were selected and submitted to the GTDB-Tk version 1.3.0 [20] for classification. Metagenome-assembled genomes (MAGs) were selected only if they contained at least one near-complete 16S rRNA or McrA gene (S1 Table). Phylogenomic trees were reconstructed by the IQ-TREE web server version 1.6.12 based on the concatenated amino acid alignment of 122 conserved archaeal proteins of the selected genomes generated by the CheckM application implemented in GTDB-Tk.

### Phenotypic characterization

#### Morphology and physiology

Determination of morphological characteristics, including ultrastructure of cells, was performed as previously described [21–23].

For the determination of the temperature range and optimum, strain J.3.6.1-F.2.7.3^T^ was cultured in DSMZ medium 1718. The same medium, supplemented with NaCl between 0 and 20 g/l, was used to determine the salinity range for growth. The pH dependence of the growth response was tested in medium 1718 prepared under a gas atmosphere of 80% N_2_ and 20% CO_2_ supplemented with formate as an electron donor. To test electron donors other than H_2_, DSMZ medium 1718 was prepared under a gas atmosphere of 80% N_2_ and 20% CO_2_, while the requirement for an organic carbon source was determined in medium without acetate. The use of alcohols as substrates was tested at a concentration of 40 mM, while other substrates were tested at a concentration of 20 mM. The growth response was recorded after the third transfer under the same conditions. The sensitivity of *Methanospirillum* cultures to ammonium was tested in DSMZ medium 1718 supplemented with 5 mM, 15 mM or 25 mM NH_4_Cl as the sole nitrogen source. The growth response was determined by measuring the optical density at 600 nm in a Genesys 30 spectrophotometer (ThermoFisher Scientific, Dreieich, Germany) equipped with a test tube holder. In addition, the methane formed during growth was measured by gas chromatography with a flame ionization detector according to a previously published protocol [1].

#### Chemotaxonomy

Analyses of intact tetraether lipids in whole cells was performed according to the method of Hopmans et al., 2000 [24]. In order to remove the headgroups from the intact polar lipids (IPLs) and to obtain the remaining core lipids, the lyophilized biomass was hydrolyzed with HCl/methanol (1.5 N) by refluxing for 3 hours. The hydrolysate was adjusted with aqueous KOH to pH 5, extracted three times with dichloromethane, and dried over Na_2_SO_4_. The hydrolyzed biomass extracts were dissolved in a solvent mixture of hexane and isopropanol in a 99:1 volume ratio. The resulting solution was then filtered through 0.45 μm PTFE filters. The filtered samples were analyzed using an Agilent 1100 HPLC-system coupled with an atmospheric pressure chemical ionization mass spectrometer (APCI/MS) and an automatic injector. Data collection and analysis were performed using the HP-Chemstation software. This analysis was conducted according to the protocols previously published [24] with the following modifications: Separation was achieved in a normal phase with two Prevail Cyano columns in series (150 mm × 2.1 mm; 3 μm) with a starting eluent of hexane:propanol (90:10, v:v) and a gradient as described in [25]. The injection volume was 10 μL. An external standard of archaeol:acyclic glycerol dialkyl tetraether (1:1, w:w) was analyzed in three concentrations to produce a standard curve for quantification of archaeol (AR) and acyclic glycerol dialkyl tetraether (GDGT-0). This standard mix was produced by isolation of the two compounds from archaeal biomass by preparative HPLC, and subsequent acid hydrolysis to remove the polar head groups. For the detection of IPLs, lyophilized biomass was extracted using a modified Bligh-Dyer procedure and analyzed using ultra-high performance liquid chromatography—high-resolution mass spectrometry (UHPLC-HRMS) as described previously [26]. IPLs were identified based on their accurate mass and on comparison of their MS^2^ spectra with published reports [25, 27].

The presence of respiratory isoprenoids in cell membranes was analyzed using protocols based on methods published previously [28, 29]. Briefly, 20–30 mg of biomass was resolved in 1 ml hexane/methanol (1 : 1) and stirred for 10 min. The non-polar phase was collected and the polar phase was re-extracted with hexane. The combined non-polar phase was loaded onto a hexane pre-equilibrated Silica Chromabond column (1 ml, Macherey-Nagel, Düren, Germany). The column was washed with hexane and the combined fraction of quinones was eluted with 10 % (v/v) methyl-tert-butyl ether in hexane. The samples were analyzed on an Acquity UPLC BEH C18 analytical column (2.1 x 100 mm, particle size 1.7 μm; Waters) with a gradient of A (isopropanol, 0.1% formic acid, 1% water) and B (acetonitrile, 0.1% formic acid), from 10% A to 60% A using an Agilent HPLC-QTOF system (positive mode, 6545 equipped with an electron spray interface and a 1290 HPLC system equipped with a diode array detector (DAD), Agilent, Santa Clara, CA, USA). The resulting chromatograms were scanned for distinct DAD absorption spectra, fragments and exact masses of mena- and ubiquinones as well as methanophenazine [30]. Additionally, eluting compounds were scanned for typical mass spectral patterns of isoprenoid compounds.

#### Genome sequencing and comparative genome analyses

Sequencing of the complete genome of J.5.4.2-T.3.5.2^T^ was performed using a hybrid sequencing strategy that included long-read PacBio and short-read Illumina sequencing technologies as previously reported [9]. The draft genome of *Methanospirillum hungatei* SK (= DSM 3595) was determined on the Illumina platform using the Nextera XT DNA Library Preparation Kit (Illumina, San Diego, USA) with modifications according to Baym *et al*. [31]. Sequencing was performed on an Illumina NextSeq^TM^ 500 with 300 cycles in 2 × 150 bp mode and assembled into contigs using SPAdes v. 3.14 [32]. The final assembly of the DSM 3595 draft genome resulted in 72 contigs with a coverage of 100x (S2 Table).

For phylogenomic analyses, average nucleotide identity (ANI) values were determined using the ANI calculator [33] and digital DNA-DNA hybridizations (dDDH) were performed using the Genome-to-Genome Distance Calculator (GGDC version 3.0) [34]. The integrated prokaryotes genome and pan-genome analysis web service (IPGA v. 1.09 [35]) was used to perform whole-genome comparisons including an estimation of the core genome and pan-genome. Synteny plots of bidirectional best Blast hits between genomes were computed using the sequence-based comparative tool of the RAST server (SEED Viewer v. 2.0) [36, 37]. Metabolic pathways were reconstructed using the annotation and comparative genome analysis tools provided by the JGI IMG v.7 platform [38].

## Results and discussion

### Isolation of pure culture

The pyrite-forming mixed culture J3 was used to selectively enrich methanogenic microorganisms, which are thought to play an important role in pyrite formation. The J3 culture was originally obtained from digested sewage sludge from the wastewater treatment plant in Konstanz, Germany. This mixed culture produced methane and pyrite for more than 20 years, as did the J5 culture, which was incubated in parallel and described previously [1]. For selective enrichment of methanogenic archaea, different antibiotics were added to the cultivation media. In a first enrichment step, a variation of DSMZ medium 119 was used that did not contain complex organic components and to which 100 mg/l rifampicin was added to prevent the growth of most bacteria (medium I, see Material and Methods). Methanogenic substrates of the enrichments were 2.0 g/l sodium formate and a gas mixture of 80% H_2_ and 20% CO_2_ added at 1 bar overpressure. After an incubation period of about 2 weeks at 28°C, the growth of cells with a morphology typical of methanospirilla was detected. This enrichment culture was attempted to be purified in DSMZ medium 119 by a dilution series. However, it turned out that the last positive culture of the dilution series was still contaminated by coccoid bacteria identified as *Sphaerochaeta* sp. Members of the genus *Sphaerochaeta* are related to free-living spirochetes that exhibit low sensitivity to rifampicin [39], which may explain the presence of these bacteria in the enrichment cultures obtained. Therefore, different antibiotics were tested that could be used to selectively inhibit the growth of *Sphaerochaeta* sp. in DSMZ medium 119 without preventing the growth of methanogenic archaea. The combination of erythromycin and kanamycin at 150 mg/l each proved to be particularly effective. However, after a dilution series with these antibiotics, still very few coccoid bacterial cells were visible. Therefore, in a third step, the growth of the remaining *Sphaerochaeta* cells was completely inhibited by adding 50 mg/l tetracycline.

The purity of the culture was then confirmed by cultivation in complex media under oxic and anoxic conditions. No growth was detected after anaerobic incubation in Wilkins-Chalgren Broth (https://mediadive.dsmz.de/medium/339a) under 80% N_2_ and 20% CO_2_ gas atmosphere and on Columbia Blood Agar plates (https://mediadive.dsmz.de/medium/429) incubated under air atmosphere. The absence of bacteria was also confirmed by a negative PCR reaction with the combination of the forward primer 10-30F (5´GAG TTT GAT CCT GGC TCA G) [40] and the reverse primer 1390R (5´CGG TGT GTA CAA GGC CC) [41] targeting the bacterial 16S rRNA gene. Direct sequencing of a partial 16S rRNA gene amplified with a primer set designed for most Archaea (ARC-8F 5´TCC GGT TGA TCC TGC C [42] and 1500Ra 5´AAGGAGGTGATCCAGCC [43]) obtained a clean sequence that could be affiliated with representatives of the genus *Methanospirillum*. The new isolate was designated J.3.6.1-F.2.7.3^T^. Upon further cultivation, the growth response of the new isolate on DSMZ medium 119 proved to be unstable. Apparently, growth was inhibited by high concentrations of complex organic nutrients, so a nutrient-poor medium with acetate as the sole source of organic carbon was developed (medium II, see material and methods).

### Ecology and evolution

#### Environmental relevance

In clone libraries of partial archaeal 16S rRNA genes obtained from a pyrite-forming enrichment culture (J5) incubated in parallel to the mixed culture J3 [1], only sequences identical to the 16S rRNA gene of the new isolate in the overlapping region of 752 nucleotides were obtained. Therefore, it can be assumed that this isolate plays a key role in the production of methane during pyrite formation. The most closely related cultured isolates were strains GP1 and SK, currently assigned to the species *M*. *hungatei* [44, 45], and the incompletely described *Methanospirillum* strain T_5_3BJ [46]. These strains had 16S rRNA gene identity values greater than 99.7% with strain J.3.6.1-F.2.7.3^T^ and were isolated from anoxic freshwater sediments or laboratory-scale anaerobic reactors designed for digestion of agro-industrial waste (S3 Table). Laboratory-scale reactors designed for the fermentation of organic waste appear to provide optimal conditions for the propagation of *Methanospirillum* strains closely related to the new isolate, as shown by several studies in which nearly identical 16S rRNA gene sequences were detected using cultivation-independent methods (S3 Table). Examples include experimental setups of digesters fed with long-chain fatty acids [47], propionate [48], or butyrate [49]. A common feature of these methanogenic enrichments is that the major substrates are fatty acids of various lengths, which are syntrophically degraded by microbial consortia. This means that the H_2_ used by methanogenic archaea as an electron donor is produced by the fermenting bacteria and only available at low concentrations [50]. The methanogenic degradation of medium and long-chain fatty acids is further complicated by the negative effect of these compounds on the proliferation of methanogenic Archaea [51]. In addition, numerous sequences representing this clade were detected in anoxic sediments of the Indian continental shelf or a river in Japan (S3 Table), habitats that probably represent a natural reservoir of this species.

**Fig 1:**
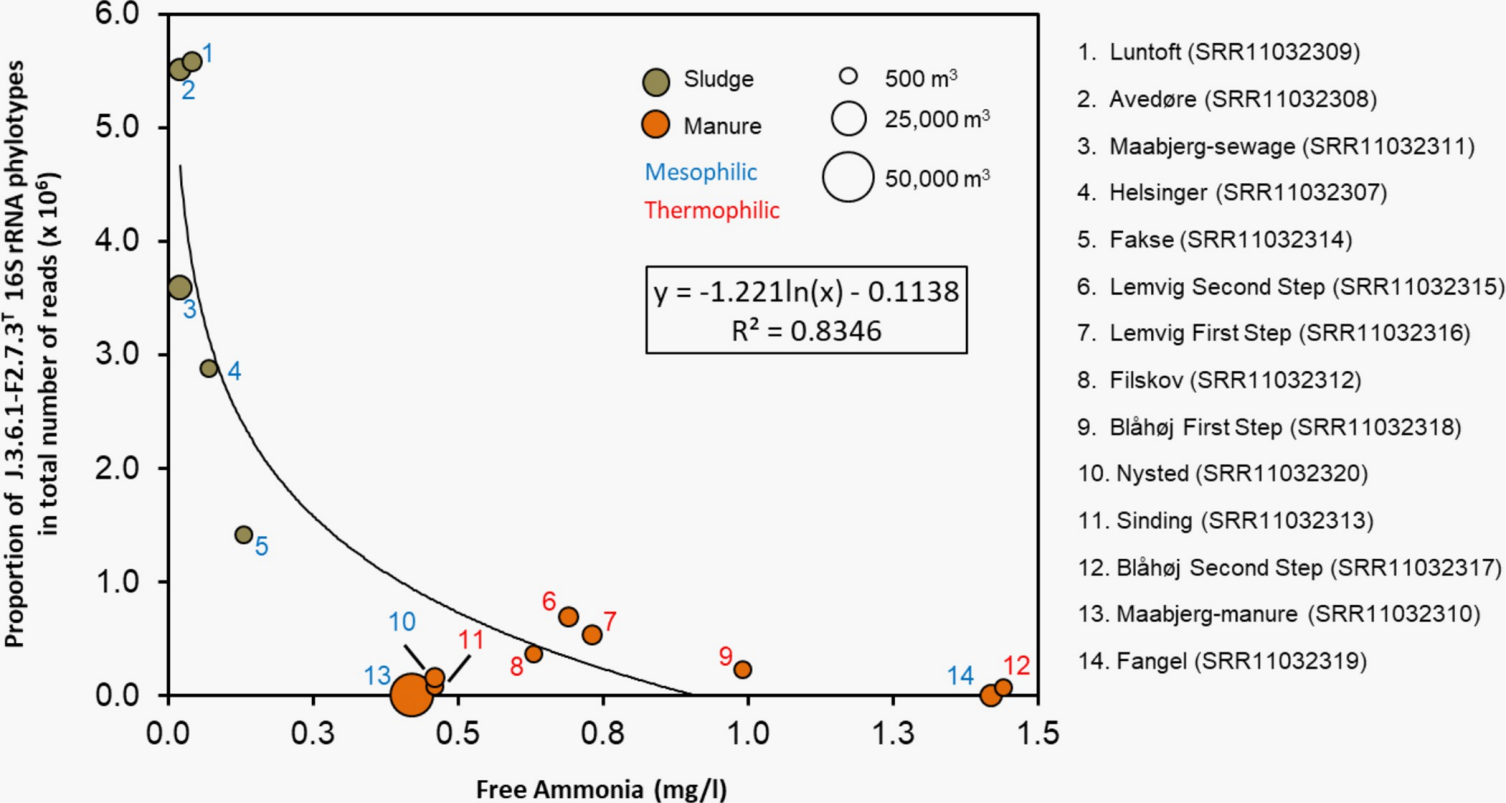
Correlation between the concentration of free ammonia and the abundance of the 16S rRNA phylotype of J.3.6.1-F.2.7.3^T^ in anaerobic digesters of twelve different full-scale biogas plants. The size of the digesters is represented by the corresponding size of the filled circles, and the location of the biogas plant is indicated by numbers. The main substrate fed and the temperature regime are indicated by colors. The best correlation of the measured values was obtained with logarithmic regression. The formula of the trend line and the coefficient of determination (R^2^) of the model are given in the graph. The data used were previously published by Campanaro *et al*. [53].

Population analyses based on amplified 16S rRNA gene sequences may be biased by differential efficiency of the PCR reaction and PCR primers used. Therefore, we searched for fragments of the 16S rRNA gene representing the new isolate in non-amplified metagenomes that are not subject to this bias. Identical or almost identical 16S rRNA gene fragments (99–100% identity) were detected in metagenomes of an anaerobic digester of a wastewater treatment plant in Germany [52] and several full-scale biogas plants in Denmark [53]. In the latter study, the process parameters in anaerobic digesters of twelve different biogas plants were described in detail. The extensive data set obtained allows interesting conclusions to be drawn about the preferred ecological niche of this clade of *Methanospirillum* strains. The highest numbers of positive hits in a standard BLAST search were found in metagenomes from biogas plants fed with domestic sewage (*e*.*g*., Luntoft), while very few, if any, sequences identical to the 16S rRNA phylotype of J.3.6.1-F.2.7.3^T^ were detected in samples from plants fed with manure such as Fangel. In a subsequent correlation analysis, we found that the frequency of this phylotype is negatively influenced by high concentrations of free ammonia, total ammonia-nitrogen or high pH values. The highest coefficient of determination was obtained for a decreasing abundance of this phylotype with increasing concentrations of free ammonia (R^2^ = 0.83, logarithmic regression type; Fig 1). Other environmental factors such as temperature, concentration of volatile fatty acids (VFA), size of the reactor or biogas production played only a minor role. It follows that the accumulation of nitrogen compounds caused by the high nitrogen load of the manure fed, ultimately leading to increased ammonia concentrations, probably prevents the efficient proliferation of this phylotype in biogas plants. Due to the complex composition of the wastewater and the high substrate turnover in the activated sludge, it is difficult to say which compounds or combination of factors are directly responsible for the observed inhibition. Interestingly, a high frequency of this *Methanospirillum* clade in biogas plants always correlated with a high abundance of acetoclastic methanogens assigned to the genera *Methanosaeta* and *Methanothrix* [53]. On the other hand, at high ammonia concentrations, there appears to be an enrichment of members of the genera *Methanosarcina* and *Methanoculleus*, which predominantly perform a hydrogenotrophic pathway of methanogenesis. The differential sensitivity of acetoclastic and hydrogenotrophic methanogens to ammonia in anaerobic digesters is well known [54] and could be a further indication of the sensitivity of this *Methanospirillum* clade to certain nitrogen compounds.

High numbers of the 16S rRNA phylotype of J.3.6.1-F.2.7.3^T^ (> 100 pos. hits) were also retrieved from metagenomes of laboratory-scale reactors set up to produce biogas from manure supplemented with long-chain fatty acids [55], but the ammonia concentrations in these laboratory-scale reactors were not reported. The relatively high abundance of this *Methanospirillum* clade in these reactors allowed in a subsequent study [56] the retrieval of a metagenome-assembled genome (AS06rmzACSIP_358, NCBI accession number JAAYBU000000000) that belongs to the same species as strain J.3.6.1-F.2.7.3^T^ according to the GTDB (GTDB 08-RS214. Available online: https://gtdb.ecogenomic.org/, accessed on 01.07.2023).

In summary, the cultivation-independent surveys of the distribution and abundance of this clade indicate an important role in the syntrophic degradation of metabolites produced, for example, during the fermentation of dead biomass. Consistent with this assumption is a recently published study showing that *M*. *hungatei* stimulates the anaerobic degradation of dead cells by bacterial scavengers, probably by preventing the accumulation of hydrogen [57]. A process that is also prevalent in laboratory-scale digesters and may even play a role in the pyrite-forming batch cultures analyzed. Phenotypic traits that may provide a selective advantage over other methanogens in these environments may include the ability to fix molecular nitrogen, utilization of very low hydrogen concentrations and a low sensitivity to the inhibitory effects of free fatty acids.

#### Phylogeny

Single-gene maximum-likelihood phylogenetic trees based on sequences of 16S rRNA genes revealed the affiliation of the novel isolate to the family *Methanospirillaceae* within the order *Methanomicrobiales* (S1 Fig). The most closely related type strains were *M*. *hungatei* JF-1^T^ and *M*. *stamsii* Pt1^T^ with 16S rRNA gene sequence identity values of 98.84 and 98.15%, respectively. The closest related cultured isolates were the strains SK and GP1, currently classified as *M*. *hungatei* with sequence identity values of 100.00 and 99.93%, respectively. The complete genomes of J.3.6.1.-F.2.7.3^T^, *M*. *hungatei* JF-1^T^ and GP1 each contain four copies of a complete rRNA operon. Multiple copies of 16S rRNA genes in the same genome are nearly identical, with only one or two base exchanges.

The reconstructed topology of the 16S rRNA tree agrees well with phylogenetic calculations based on deduced amino acid sequences of the methyl-coenzyme M reductase alpha subunit McrA (S1 Fig). McrA is part of an enzyme that catalyzes the last step of methanogenesis, namely the production of methane by the reduction of methyl-coenzyme M with coenzyme B [58]. McrA is therefore often used as a phylogenetic marker of methanogens [59]. However, the use of McrA to infer phylogenies is complicated by the presence of two isoenzymes in numerous species of methanogens [60]. The amino acid sequence difference between the two isoenzymes of the same strain may be greater than that between enzymes from species belonging to phylogenetically distantly related groups. Possible reasons for deviating sequences of McrA in some methanogens could be horizontal transfer of the encoding gene or divergent evolution after gene duplication. Therefore, the sequences of both isoenzymes were used for the McrA tree only if they belong to the same phylogenetic group, as is the case, for example, in the genus *Methanoculleus*. However, there are also methanogens that contain only one copy of the *mcrA* gene, such as strain J.3.6.1.-F.2.7.3^T^. The amino acid identities to the McrA proteins of the most closely related type strains *M*. *hungatei* JF-1^T^ and *M*. *stamsii* Pt1^T^, which also have only one copy of the encoding gene, are 97.00 and 96.83%, respectively. Remarkably, strain *M*. *hungatei* GP1 is an exception within this group because it contains two divergent copies of the *mcrA* gene. One of the two respective isoenzymes (WP_218607010) has an identity value of 99.29% with McrA of J.3.6.1.-F.2.7.3^T^, while the isoenzyme (WP_218608473) has an amino acid identity of only 76.54%, indicating a possible horizontal transfer of the encoding gene. A partial sequence of McrA (261 amino acids) from the incompletely described *Methanospirillum* strain T_5_3BJ (MT551924) is identical to McrA of J.3.6.1.-F.2.7.3^T^, confirming the result of the 16S rRNA comparison (S3 Table). Both suggest a close phylogenetic relationship at species level.

**Fig 2:**
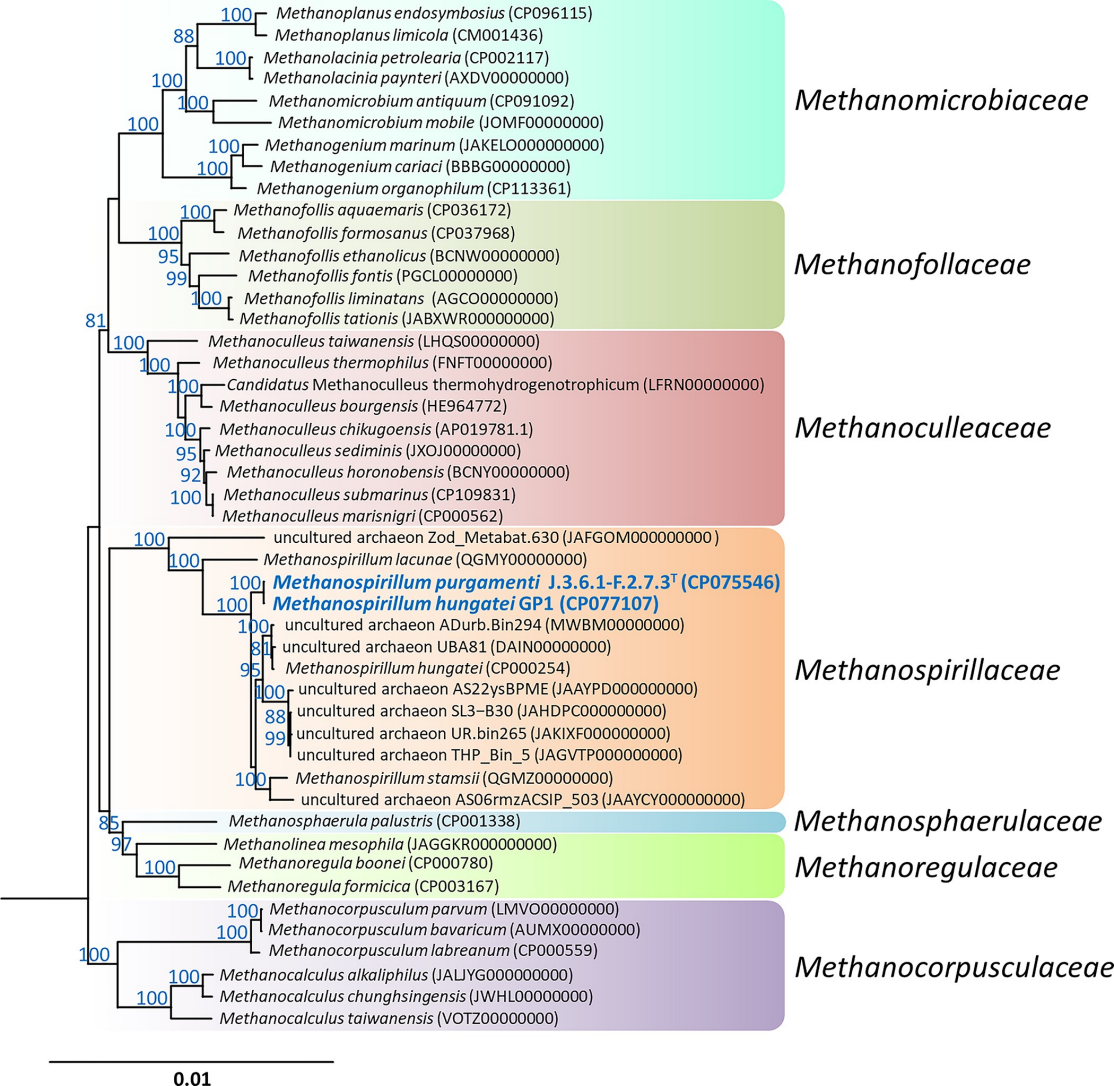
Phylogenomic tree based on concatenated amino acid sequences of 122 conserved archaeal proteins showing the placement of the strain J.3.6.1-F.2.7.3^T^ within the order *Methanomicrobiales*. The tree was reconstructed under the maximum-likelihood criterion with the model LG+F+I+G4 of protein evolution and ultrafast bootstrap analysis with a maximum of 1,000 iterations. The genome of *Methanocella paludicola* (AP011532, not shown) was used as an outgroup. The names of the representative strains of each species are listed in S1 Table. Accession numbers are given in parenthesis. Only bootstrap support values above 80% are shown at the respective nodes. Background shading with different colors is used to delineate clades at the family level. The scale bar indicates the expected number of substitutions per site.

In addition to trees based on individual genes, genome-based phylogenetic calculations were used to elucidate the phylogenetic framework within the order *Methanomicrobiales*. Fig 2 shows a tree based on the concatenated alignment of the amino acids of 122 proteins conserved in Archaea. The type strains of *M*. *hungatei* and *M*. *stamsii*, together with J.3.6.1-F.2.7.3^T^, form a common branch within the *Methanospirillaceae* that is distinct from the *M*. *lacunae* lineage. The species *M*. *lacunae* [61] and *M*. *psychrodurum* [62] also differ from the other species isolated from wastewater in terms of their preferred habitat, namely moist soils. In addition to the family *Methanospirillaceae*, six other monophyletic groups are recognizable and can be defined as families when rank normalization is applied, as proposed by the GTDB [63]. Recently, attempts have been made to apply this concept throughout taxonomy, resulting in the official proposal of the new families *Methanofollaceae*, *Methanoculleaceae*, and *Methanosphaerulaceae* [64].

#### Classification

Our phylogenetic analyses revealed that the new isolate J.3.6.1-F.2.7.3^T^ is most closely related to the two type strains *M*. *hungatei* JF-1^T^ and *M*. *stamsii* Pt1^T^. Three other available isolates, *M*. *hungatei* SK, *M*. *hungatei* GP1, and *Methanospirillum* sp. T_5_3BJ, and an uncultured archaeon represented by MAG AS06rmzACSIP_358, are also closely related. Based on the position in the phylogenetic tree and the high 16S rRNA identity with the type strain of the type species of *Methanospirillum*, *M*. *hungatei*, the new isolate can be unambiguously assigned to the genus *Methanospirillum*. However, genome comparisons are required for further classification at the species level.

Established genome-based tools for species demarcation are based on the determination of genome-wide average nucleotide identity (ANI) or digital DNA-DNA hybridization (dDDH). Widely accepted thresholds for species delineation are 95% for ANI and 70% for DDH [65]. Genomes of the two most closely related type strains *M*. *hungatei* JF-1^T^ and *M*. *stamsii* Pt1^T^ have ANI values of 82.8 and 80.2%, respectively, with the genome of J.3.6.1-F.2.7.3^T^, well below the cut-off value of 95%. The respective dDDH values were correspondingly low at 21.9 and 19.5%. It follows that strain J.3.6.1-F.2.7.3^T^ represents a new species within the genus *Methanospirillum*. The distant position of *M*. *hungatei* GP1 to the type strain JF-1^T^ in the reconstructed phylogenetic trees was confirmed by the obtained ANI and digital DDH values of 82.3 and 21.6%, respectively. On the other hand, ANI and dDDH values of 98.4% and 85.4% were obtained with the new isolate J.3.6.1-F.2.7.3^T^, respectively, so that both strains can be assigned to the same species. The same is true for the strain *M*. *hungatei* SK and the MAG AS06rmzACSIP_358 for which however only incomplete draft genomes are available. Table 1 lists the ANI and dDDH values obtained from a pairwise comparison of the genomes of representatives of this clade of *Methanospirillum* strains with the most closely related type strains. Unfortunately, no genome sequence is available from isolate T_5_3BJ, but based on the high identity values of the 16S rRNA and McrA, it can be assumed that this strain can be assigned to the same taxon as J.3.6.1-F.2.7.3^T^.

**Table 1:** Matrix of average nucleotide identity (ANI) and digital DNA-DNA hybridization (dDDH) values between genomes of *Methanospirillum* strains related to the isolate J.3.6.1.-F.2.7.3^T^.

	J.3.6.1-F.2.7.3^T^	SK	GP1	AS06rmzACSIP_358	JF-1^T^	Pt1^T^	Ki8-1^T^
*M*. *purgamenti* J.3.6.1-F.2.7.3^T^	-	**100.0**	**85.4**	**85.4**	21.9	19.5	18.6
*M*. *hungatei* SK	**100.0**	-	**85.3**	**85.5**	21.7	19.3	17.9
*M*. *hungatei* GP1	**98.4**	**98.4**	-	**88.6**	21.6	19.6	19.1
*M*. sp. AS06rmzACSIP_358	**98.3**	**98.3**	**98.7**	-	22.7	20.5	16.9
*M*. *hungatei* JF-1^T^	82.8	82.5	82.3	81.7	-	19.3	18.3
*M*. *stamsii* Pt1^T^	80.2	80.0	80.3	80.0	79.5	-	17.5
*M*. *lacunae* Ki8-1^T^	76.5	76.5	76.9	75.1	76.5	76.5	-

The upper right triangle indicates the dDDH values and the lower left triangle pairwise ANI values (%). Values above the respective thresholds for species delineation (>95% for ANI and >70% for dDDH) are highlighted in bold.

Consequently, four pure cultures and one MAG obtained mainly from anoxic wastewater can be assigned to a new taxon distinct from the closely related species *M*. *hungatei* and *M*. *stamsii*. This highlights the importance of this taxon in engineered environments, especially anaerobic bioreactors. To reflect the preferred ecological niche of the representatives of this taxon, we propose the name *M*. *purgamenti* sp. nov. (*purgamenti* means from sewage). A formal description is presented at the end of this article.

### Characterization of the phenotype

#### Morphology

**Fig 3:**
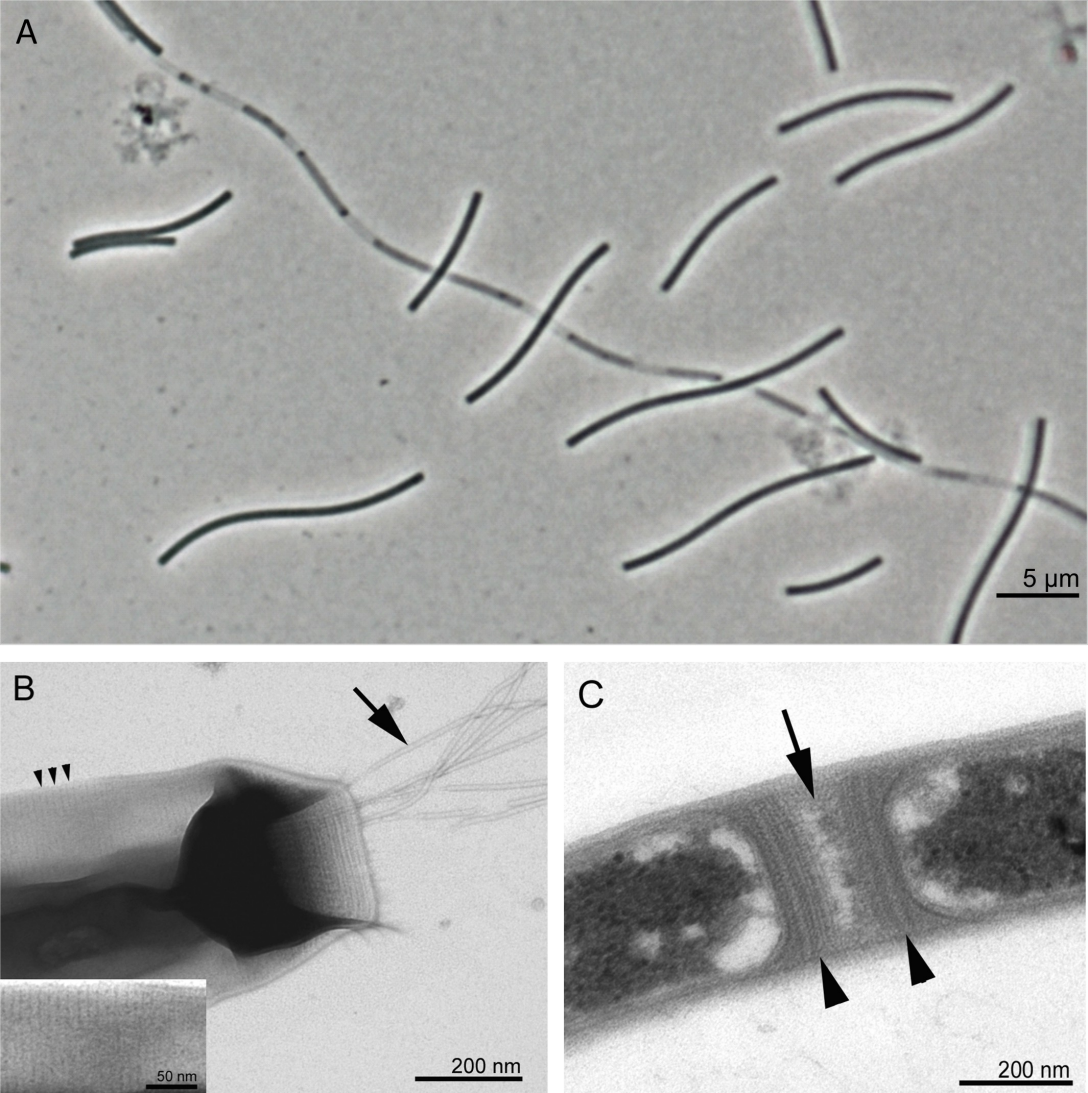
Morphology of cells of the isolate J.3.6.1-F.2.7.3^T^. (**A**) Phase contrast micrograph of a culture in the stationary phase showing several single cells and a long filament containing partially lysed cells; (**B**) negative contrast transmission electron micrograph of the end of a whole cell with a bundle of polar archaella (large arrow), the spacing of the striation on the sheath surface is marked with small arrowheads (see also the inset with an enlarged section); (**C**) negative contrast transmission electron micrograph of a thin section showing the cell spacer (large arrow) and the multilayered spacer plugs (arrowheads).

Cells of strain J.3.6.1-F.2.7.3^T^ are slender, curved rods with blunt ends that have an average length of 10 μm (range 7–20 μm) and a width of 0.4–0.5 μm. The Gram reaction of individual cells is negative. The cells are enclosed in a wavy sheath-like structure, which usually contains multiple cells and can reach a length of more than 100 μm. In older cultures, partial cell lysis reveals the arrangement of cells within the filaments, and sometimes dark inclusion bodies are visible at both cell poles (Fig 3A). In the related strain *M*. *hungatei* JF-1 similar inclusion bodies were identified as polyphosphate granules that can be used for the storage of energy and phosphorous [66]. Endospores were not detected. A slightly meandering motility is conferred by polar tufts of archaella (Fig 3B). The structure of the tubular sheath was examined in more detail using transmission electron microscopy and it was found that there are great similarities to that of other *Methanospirillum* strains [67, 68]. In negatively stained samples, transmission electron micrographs reveal a characteristic striated outer surface of the sheath tubes, possibly due to stacked hoops as structural elements. The striation has around 13 nm spacings, which corresponds to the distance between individual rings constituting the sheath [69]. Individual cells are separated within the filaments by cell spacers bounded by multilaminar rectangular structures known as spacer plugs (Fig 3C).

#### Physiology

The temperature range of strain J.3.6.1-F.2.7.3^T^ was determined to be 25–40°C with an optimum at 35°C. This result agrees well with data reported for *Methanospirillum* strains SK and GP1, which have a temperature range for growth of 20–41°C and 25–45°C, respectively, and a temperature optimum at 35–37°C [44, 70]. In contrast, the type strain of *M*. *hungatei* is adapted to higher temperatures, with a growth range between 30 and 50°C and an optimum at 40–45°C [44]. On the other hand, the closely related species *M*. *stamsii* prefers lower temperatures with a growth range between 5 and 37°C and an optimum at 20–30°C [71]. In addition, strain J.3.6.1-F.2.7.3^T^ was found to be able to grow at pH values between 5.5 and 7.5, with an optimum at pH 7.0–7.2 and at a salinity of 0–15 g/l NaCl (optimum 3 g/l NaCl). Under optimal conditions, a maximum growth rate of 0.03 h^-1^ was obtained. The corresponding generation time of 10 hours is significantly shorter than the values reported for the type strains of *M*. *stamsii*, *M*. *lacunae* and *M*. *hungatei* [71], but is similar to the determined value of strain SK [70].

Vitamins, 2-mercaptoethanesulfonic acid (coenzyme M), fatty acids, or complex nutrients were not required for growth. However, acetate was essential as a carbon source in addition to CO_2_. Interestingly, it was found that the addition of sludge fluid (50 ml/l), yeast extract and Trypticase peptone (1 g/l each) did not have a stimulatory effect on the growth of strain J.3.6.1-F.2.7.3^T^. This is consistent with previous observations in strains GP1 and SK [70, 72]. These are notable differences from the type strain of *M*. *hungatei*, which shows best growth in complex media containing yeast extract and Trypticase peptone, but does not require acetate as an organic carbon source [73]. These results are consistent with the cultivation-independent data shown in Fig 1, which indicate inhibition of this *Methanospirillum* clade at sites with particularly high concentrations of nitrogenous compounds from manure. Under laboratory conditions, strain GP1 was inhibited only by ammonium concentrations above 5 mM in batch cultures, whereas limiting amounts of ammonium had no positive effect on growth [72]. In order to compare the nitrogen sensitivity of the new isolate with that of the type strain of *M*. *hungatei*, batch cultures of both strains were incubated in mineral medium supplemented with different amounts of ammonium chloride as nitrogen source. It was found that no significant inhibition of growth or methane production was detectable under the selected experimental conditions in the range between 5 and 25 mM NH_4_Cl. It is therefore questionable what explains the inhibition of this *Methanospirillum* clade in environments rich in complex nutrients. One possibility is that the inhibition in the natural environment could be caused by some by-products of the anaerobic nitrogen metabolism.

In the presence of acetate as an organic carbon source, formate and isopropanol (2-propanol) can be used as alternative electron donors instead of hydrogen for methanogenesis by strain J.3.6.1-F.2.7.3^T^. No growth was observed with formate or 2-propanol without acetate, indicating that both substrates were used only as hydrogen donors. Interestingly, it was found that strains SK and GP1 were also able to utilize 2-propanol in the presence of acetate as an organic carbon source, whereas strain *M*. *hungatei* JF-1^T^ was not [45]. This characteristic is therefore suitable for distinguishing between the two species. Furthermore, the ability to utilize 2-propanol may reflect adaptation to a syntrophic lifestyle, as it is a fermentation product of some anaerobic bacteria, *e*.*g*. *Clostridium beijerinckii* [74]. The alternative electron donors trimethylammonium chloride, acetate, pyruvate, methanol, ethanol, or 1-propanol could not be used with or without acetate as carbon source, indicating a metabolic specialization common among *Methanospirillaceae*.

#### Chemotaxonomy

The gram-negative cell walls of representatives of the genus *Methanospirillum* are composed of a cytoplasmic membrane surrounded by an outer protein layer consisting of regularly arranged protein subunits [75]. No pseudomurein sacculus is present [76], but one to several cells are enclosed in a tubular sheath structure. Amino acid compositions of the structural proteins of the sheath were previously analyzed in detail, and significant differences were found between the strains *M*. *hungatei* JF-1^T^ and GP1. For instance, the ratio of acidic to basic amino acids differed between the two strains, reaching values of 2.87 and 4.37, respectively [77].

For this study, the core lipid and intact polar lipid inventory of the novel isolate was analyzed and compared with type strains of the related *Methanospirillum* species *M*. *hungatei*, *M*. *stamsii* and *M*. *lacunae*. The core lipids detected were relatively similar in all four strains. A standard acyclic tetraether (GDGT-0) was the dominant core lipid in all strains (59–61%), followed by dialkyl glycerol diethers (archaeols) as major component (37–41%). Glycerol trialkyl glycerol tetraether (GTGT-0, [78]) was also detected as a minor component (0.5–2%), but no intact polar lipids based on GTGT-0 were identified. An extensive array of GDGT-0- and AR-based IPLs could be determined in the *Methanospirillum* strains studied. Most of the structures identified were previously described in studies using the *Methanospirillum* strain GP1 [79]. The relative abundance of the detected AR and GDGT-0 IPLs in all four strains is shown in Table 2. It should be noted that IPL species have diverse degrees of ionization efficiencies and hence the peak areas, in response units, of different components do not necessarily reflect their actual relative abundance. However, this method allows for comparison between samples when analyzed together. The dominant polar head group of the AR-IPLs of all strains was aminopentanetetrol (APT), with differing degrees of N-methylation (Table 2). Some of them contained AR with one to three unsaturations. In addition, one or two sugar units as well as phosphoglycerol were detected as polar head group moieties. Archaeol without a polar head group was also detected and occurred in significant amounts in strain *M*. *lacunae* Ki8-1^T^. The GDGT-0-based IPLs had a similar distribution of polar head groups as the AR IPLs. Two types were identified that contained both a disaccharide and a N,N,N-trimethyl APT or N,N-dimethyl APT head group, corresponding to the tetraether phosphoglycolipids PGL-III/IV or PGL-VI/VII identified previously in *Methanospirillum* strain GP1 [80]. The determined IPL profiles were in good agreement with the phylogenetic positions of the analyzed strains supporting the assumption that the IPL distribution represents a useful characteristic for the classification of Archaea [80, 81]. The IPL profile of the type strain of *M*. *lacunae* can be reliably distinguished from the other *Methanospirillum* strains and is characterized by divergent amounts of unmodified archaeol, unsaturated archaeols, and 2Gly-GDGT-0. In contrast, the two patterns of *M*. *hungatei* JF-1^T^ and J.3.6.1-F.2.7.3^T^ are very similar and can only be distinguished by the different amounts of 2Gly, N,N,N-trimethyl APT-GDGT-0 (Table 2).

**Table 2:** Distribution patterns of intact polar lipids containing standard archaeal dialkyl glycerol diether (archaeol) or acyclic glycerol dialkyl glycerol tetraether (GDGT-0) as core lipids in the novel isolate J.3.6.1-F.2.7.3^T^ compared to type strains of related *Methanospirillum* species.

Polar head group	Core lipid	AEC	*M*. *purgamenti* J.3.6.1-F.2.7.3^T^	*M*. *hungatei* JF-1^T^	*M*. *stamsii* Pt1^T^	*M*. *lacunae* Ki8-1^T^
Diether lipids						
** **N,N,N-trimethyl APT	AR	C_51_H_107_NO_9_P	**16.3**	**20.8**	**20.1**	**12.8**
** **N,N-dimethyl APT	AR	C_50_H_105_NO_9_P	**27.8**	**25.3**	**31.6**	**33.4**
** **N,N-dimethyl APT	AR(1)	C_50_H_103_NO_9_P	**5.7**	**7.5**	**9.0**	0.5
** **N,N-dimethyl APT	AR(2)	C_50_H_101_NO_9_P	**6.3**	**5.6**	**11.5**	1.0
** **N,N-dimethyl APT	AR(3)	C_50_H_99_NO_9_P	**7.2**	**6.7**	**10.5**	3.4
** **2Gly	AR	C_55_H_112_NO_13_	**18.9**	**20.3**	0.2	**22.7**
** **2Gly	AR(1)	C_55_H_110_NO_13_	1.3	1.6	2.3	0.3
** **Gly	AR	C_49_H_102_NO_8_	2.1	1.2	2.0	2.2
** **PG	AR	C_46_H_96_O_8_P	**13.3**	**10.5**	**11.9**	**12.0**
** **None	AR	C_43_H_89_O_3_	1.1	0.5	0.9	**11.8**
Sum			100	100	100	100
Tetraether lipids						
** **2Gly	GDGT-0	C_98_H_192_O_16_	3.2	1.2	0.1	**8.4**
** **PG, 2Gly	GDGT-0	C_101_H_200_O_21_P	**76.3**	**72.0**	**74.1**	**19.7**
** **2Gly, N,N-dimethyl APT	GDGT-0	C_105_H_209_NO_22_P	**16.7**	**17.5**	**20.6**	**65.9**
** **2Gly, N,N,N-trimethyl APT	GDGT-0	C_106_H_211_NO_22_P	3.9	**9.3**	**5.2**	**6.0**
Sum			100	100	100	100

Values are percentages of total diether or tetraether lipids, respectively. Major polar lipids (>5% of the total amount) are given in bold. Abbreviations: AEC: assigned elemental composition, AR: archaeol (the number in parenthesis indicates the number of unsaturations), N,N,N-trimethyl APT: phospho-N,N,N-trimethylaminopentanetetrol, N,N-dimethyl APT: phospho-N,N-dimethylaminopentanetetrol, GDGT-0: acyclic glycerol dialkyl glycerol tetraether, 2Gly: disaccharide, Gly: monosaccharide, PG: phosphoglycerol.

Respiratory lipoquinones or methanophenazine were not detected in any of the strains studied, consistent with previous results [82]. No attempt has been made to detect cytochromes in cell extracts, but in a previous study no cytochromes were detected biochemically in *M*. *hungatei* JF-1 [83] and the corresponding genes for cytochromes *c* are absent in *Methanomicrobiales* [84].

### Comparative genome analysis

#### Genome properties

The genome of strain J.3.6.1-F.2.7.3^T^ consists of a circular chromosome of 3,524,547 bp with a G+C content of 42.1 mol%. A total of 3,271 genes were predicted, including 3,271 protein-coding genes and 71 RNA-coding genes. The genome contains four complete rRNA operons (S2 Table). These values compare well with the complete genome sequence of *M*. *hungatei* GP1 (CP077107) and the draft genome sequence of strain SK (this study). In contrast, the G+C content of the genomic DNA of *M*. *hungatei* JF-1^T^ is considerably higher (45.1 mol%), while the genome of *M*. *stamsii* Pt1^T^ contains more coding sequences. All members of this clade of *Methanospirillum* strains contain multiple CRISPR arrays, indicating a constant threat from foreign DNA, for example by infection with archaeal viruses. In addition, several restriction-modification systems were detected in the genome of J.3.6.1-F.2.7.3^T^ indicating numerous defense mechanisms. Despite the observed prevalence of transposase genes in the analyzed genomes, the integrity and stability of the genome structure seem to be efficiently maintained during evolution. Synteny plots of bidirectional best hits between the genome of J.3.6.1-F.2.7.3^T^ and closely related strains illustrate that the contigs of the draft genome of strain SK could be matched almost perfectly to the complete genome of the novel isolate J.3.6.1-F.2.7.3^T^ (S2 Fig). Together with the ANI value of 100.0%, this indicates that both isolates could represent identical strains or even clones [85] despite their different history and source of isolation. The genome of *M*. *hungatei* GP1 shows still a high degree of synteny with only a few breaks, whereas the genome of the type strain of *M*. *hungatei* shows less agreement (S2 Fig).

#### Gene content

**Fig 4:**
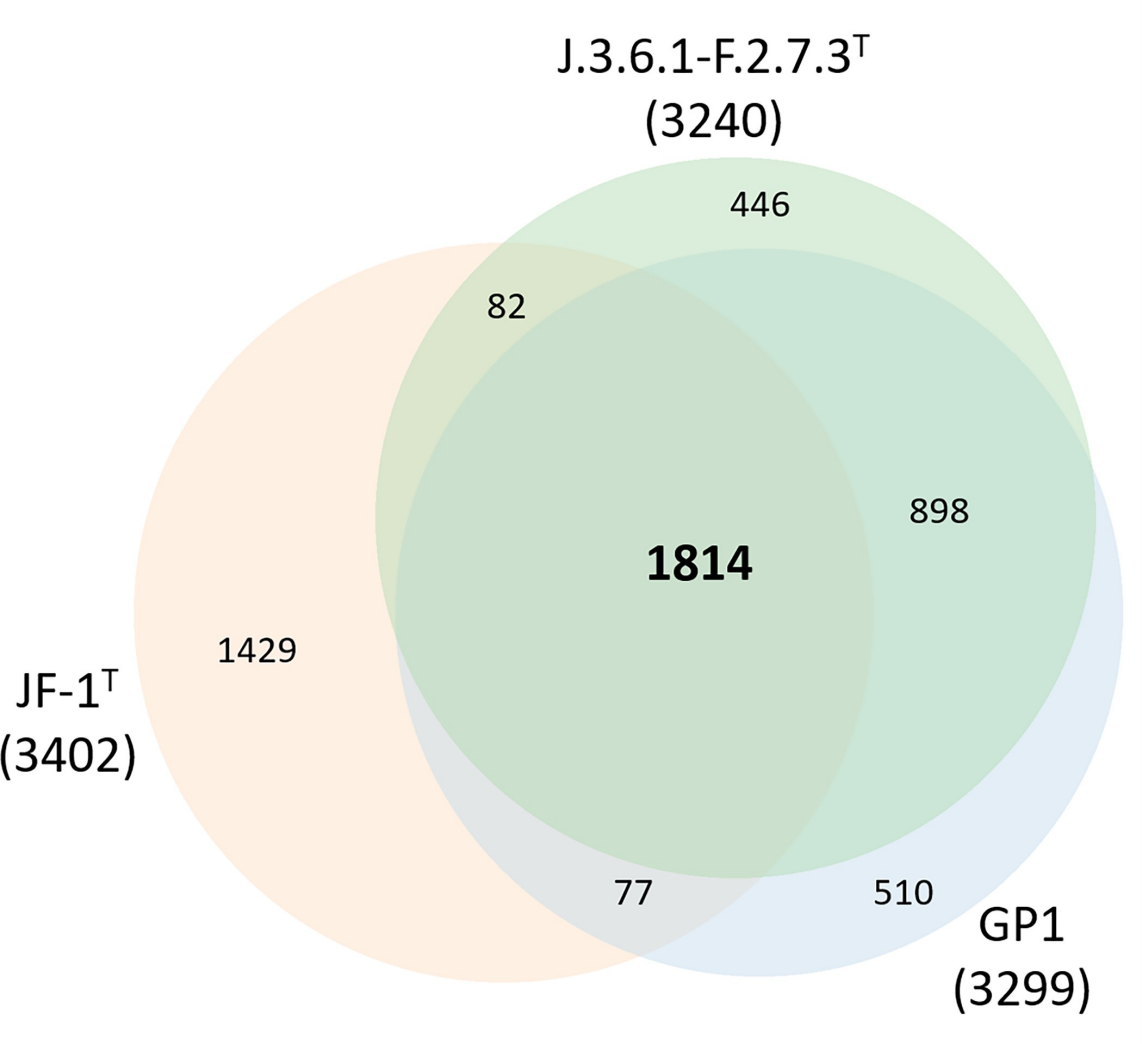
Venn diagram depicting the intersections of sets of predicted proteins of *M*. *hungatei* JF-1^T^, *M*. *hungatei* GP1 and the novel isolate J.3.6.1-F.2.7.3^T^. The corresponding protein sets and their overlaps were calculated with the Roary software tool [87], which is included in the IPGA package, using the default settings. Total numbers are in parentheses. Diagram generated using the area-proportional Venn diagram plotter from BioInforRX (https://bioinforx.com/apps/venn.php).

The pan-genome of the three isolates J.3.6.1-F.2.7.3^T^, SK and GP1, which form a clade at the species level, contains 3794 protein sets. The core genome was relatively large with 2698 protein families. Although the number of available genomes is still too small for definitive conclusions, this may indicate a closed pan-genome reflecting a distinct niche specialization and efficient control of horizontal gene transfer in this species [86]. In the Venn diagram shown in Fig 4 the difference in gene content between *M*. *hungatei* JF-1^T^, strain GP1 and J.3.6.1-F.2.7.3^T^ is illustrated. The overlap of homologous protein sets between the strains J.3.6.1-F.2.7.3^T^ and GP1 is 2712, while there is only an intersection of 1891 proteins between the type strain of *M*. *hungatei* and GP1. Accordingly, the number of proteins specific for strain JF-1^T^ is much higher (1429) than for strains GP1 (510) and J.3.6.1-F.2.7.3^T^ (446), reflecting the phylogenetic and phenotypic distance between both species.

Genes for known phenotypic traits that are present in the strains GP1 and J.3.6.1-F.2.7.3^T^ but absent in *M*. *hungatei* JF-1^T^ encode, for example, a putative F_420_-dependent secondary alcohol dehydrogenase (KHC33_06890, WP_214420977) and a nitrogenase complex (KHC33_09860–09900). The corresponding genes are also present in the genome of the *Methanospirillum* strain SK and are therefore species-specific. The observed differences in the effects of organic nitrogen compounds on growth between *M*. *hungatei* JF-1^T^ and the strains GP1, SK and J.3.6.1-F.2.7.3^T^ may be correlated with the presence of nitrogenase in these strains. In the latter *Methanospirillum* strains, the genes of the nitrogenase complex are closely linked to the genes of the CO dehydrogenase (CODH)/acetyl-CoA synthase (ACS) complex, which is the key enzyme of the reductive acetyl-CoA pathway. While the activity of nitrogenase was confirmed in laboratory experiments with strain GP1 [88], the function of the CODH/ACS complex in these strains is unclear as they are reportedly unable to use CO_2_ as their sole carbon source [44, 70]. Therefore, these strains are not capable of using the reductive acetyl-CoA pathway to maintain the intracellular redox balance, as proposed for acetogens growing on organic carbon sources [89]. Under certain circumstances, this role could be taken over by the nitrogenase complex, which, for example, is part of the redox balancing system in photoheterotrophically growing bacteria under N-limitation [90]. High N/C ratios of nutrients could lead to an inhibition of nitrogenase and therefore a redox imbalance within cells. If this hypothetical scenario is correct, *M*. *hungatei* may have adapted to ecological niches with an excess of bound nitrogen by deleting the nitrogenase complex and instead utilizing the reductive acetyl-CoA pathway to maintain a cellular redox homeostasis and autotrophic CO_2_ assimilation.

**Fig 5:**
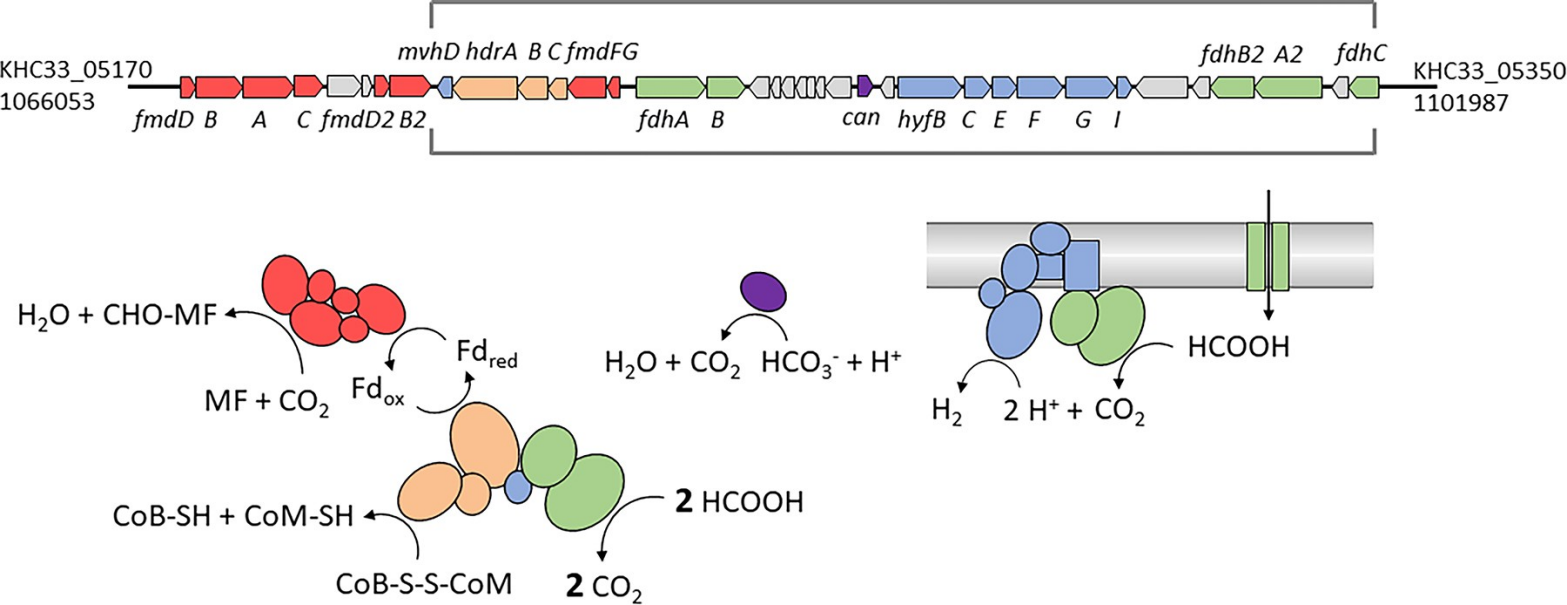
Conserved cluster of genes present in the genome of isolate J.3.6.1-F.2.7.3^T^ and related *Methanospirillum* strains with the ability to utilize 2-propanol. The locus tags are given for the first and last gene of the region found in the genome of J.3.6.1-F.2.7.3^T^. The region marked with square brackets is also conserved in *M*. *hungatei* JF-1^T^. Genes and the corresponding enzymes involved in key steps of methanogenesis are highlighted in color: red, formylmethanofuran dehydrogenase (FmdABCDFG); orange, heterodisulfide reductase (HdrABC); purple, carbonic anhydrase (Can); blue, electron-transfer subunit of F_420_-nonreducing hydrogenases (MvhD) and membrane-bound H_2_-evolving [NiFe]-hydrogenase (HyfBCEFGI); green, F_420_-dependent formate dehydrogenase (FdhAB) and formate transporter (FdhC). Abbreviations: CoB, coenzyme B; CoM, coenzyme M; F_420_, coenzyme F_420_; Fd, ferredoxin; MF, methanofuran.

The presence of the F_420_-dependent secondary alcohol dehydrogenase in *Methanospirillum* strains GP1, SK and J.3.6.1-F.2.7.3^T^ correlates with the ability of these strains to utilize 2-propanol as an electron donor [91, 92]. In addition, we were able to show that the strain *M*. *lacunae* Ki8-1^T^ (= DSM 22751^T^) encoding this gene can utilize 2-propanol after a prolonged lag phase of 3 weeks. The long time required for adaptation to this substrate may have led to the fact that 2-propanol was not identified as an electron donor in the original description [61]. Various possible pathways are currently being discussed that could enable the electron flow between the reduced F_420_ and ferredoxin required for methanogenesis [91, 93]. It is however unlikely that a H_2_-dependent N5,N10-methenyltetrahydromethanopterin hydrogenase (Hmd) is involved, because it is encoded only in strain GP1, but not the other strains using 2-propanol [91]. Currently, two possible pathways for F_420_ utilization are discussed, including either formate or 3-phosphoglycerate as intermediate [93]. A third possibility would be that F_420_H_2_ can be used as an electron donor by the heterodisulfide reductase (Hdr) directly, which in turn reduces ferredoxin and heterodisulfide by electron bifurcation, as shown for the Hdr complex in *Methanosarcina acetivorans* [94]. The genes for the Hdr complex in 2-propanol utilizing *Methanospirillum* strains are located in a conserved genomic region, which spans around 36 kb of DNA and contains also genes for a formate transporter *fdhC*, two coenzyme F_420_-dependent formate dehydrogenases (*fdhA1B1*, *fdhA2B2)*, and a putative formate-dependent membrane-associated hydrogenase (*hyfBCEFGI*) with similarities to the hydrogenase-4 of *Escherichia coli* [95]. Fig 5 shows the arrangement of the genes in this region for strain J.3.6.1-F.2.7.3^T^ (KHC33_05170–05350). A large part of this genomic region between KHC33_05210 and KHC33_05350 is also conserved in *M*. *hungatei* JF-1^T^. The *hdrABC* genes are flanked by genes encoding for the subunit D of a F_420_-nonreducing hydrogenase (*mvhD*) and formylmethanofuran dehydrogenase subunits (*fmdFG*). MvhD is important for the transfer of electrons from the F_420_-nonreducing hydrogenase (Mvh) or formate dehydrogenase to subunit A of the heterodisulfide reductase Hdr [96]. Since *Methanospirillum* strains of this clade do not encode Mvh hydrogenase [91, 97], it is likely that formate is used as an electron donor for the first and last step of methanogenesis (Fig 5). During growth with 2-propanol or H_2_, the formate required for methanogenesis could be produced from F_420_H_2_ and CO_2_ by the enzyme F_420_-dependent formate dehydrogenase. In this context, the observation that *M*. *hungatei* JF-1^T^ requires an active formate dehydrogenase for growth on H_2_ and CO_2_ is of interest [98]. Genes for additional formate dehydrogenases (Fdh3-6) were detected within the genome of strain J.3.6.1-F.2.7.3^T^ which further indicates the importance of formate as substrate in this *Methanospirillum* species and other representatives of the *Methanomicrobiales* [96]. The importance of formate as a key metabolite in many methanogenic microorganisms could suggest an early role in evolution. It has been postulated that formate has played a significant role in the evolution of this metabolic pathway under hyperalkaline conditions characterized by the limitation of dissolved inorganic carbon on the early Earth [99].

Previously, it was suggested that the utilization of 2-propanol in methanogens occurs according to the following equation [70]:

$$CO_{2}+4\;C_{3}H_{8}O\to CH_{4}+4\;C_{3}H_{6}O+2\;H_{2}O(\Delta G{}^{\circ}’=-36.5\;kJ/mol\;CO_{2})$$

**Fig 6:**
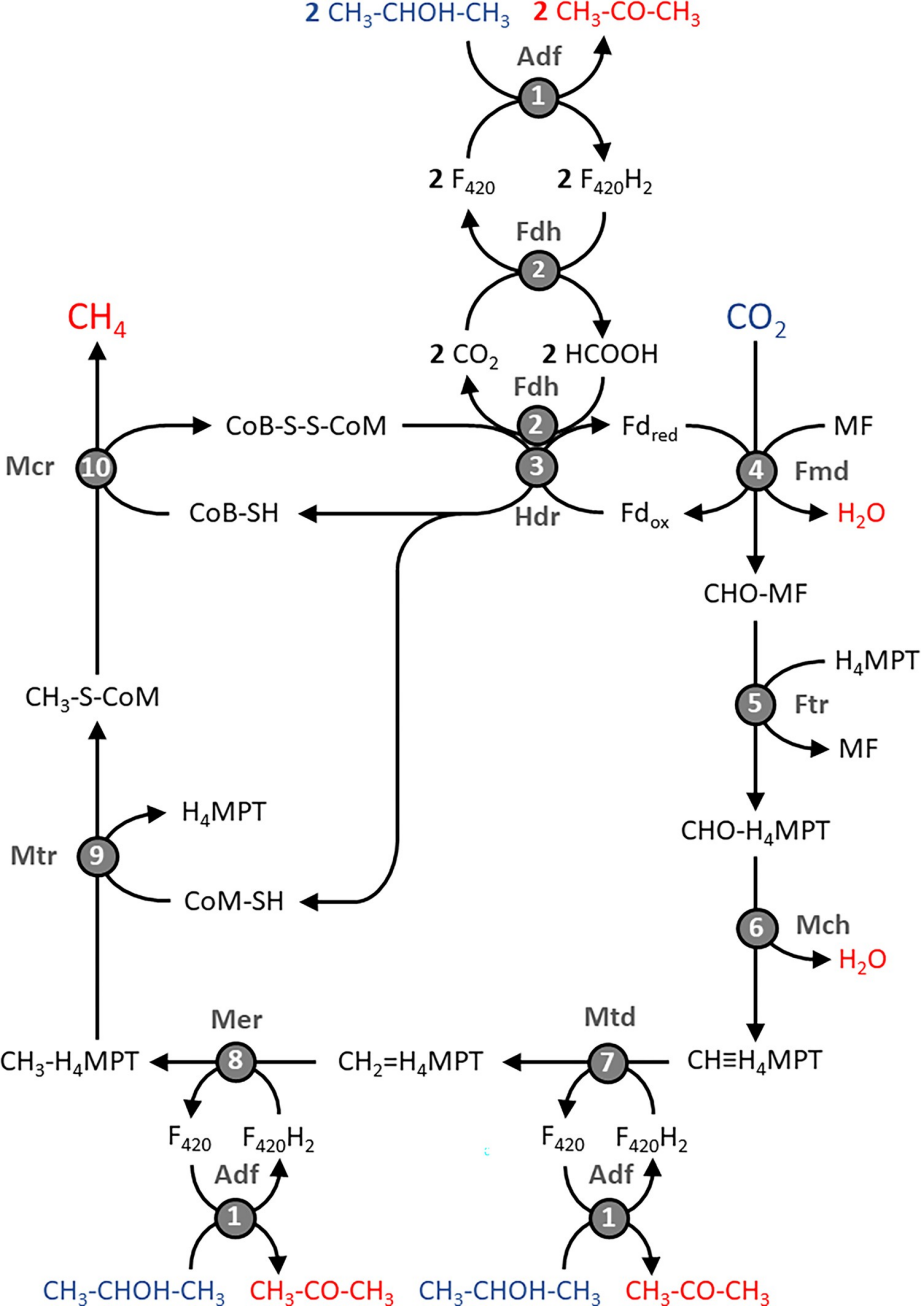
Proposed pathway for the utilization of 2-propanol in *Methanospirillum* strains. The reactants of the metabolic pathway are shown in blue and the reaction products in red. Numbers in circles indicate involved enzymes: 1, F_420_-dependent secondary alcohol dehydrogenase (Adf); 2, F_420_-dependent formate dehydrogenase (Fdh); 3; heterodisulfide reductase, bifurcating (Hdr); 4, formyl-MF dehydrogenase (Fmd); 5, formyl-MF:H_4_MPT formyltransferase (Ftr); 6, methenyl-H_4_MPT cyclohydrolase (Mch); 7, methylene-H_4_MPT dehydrogenase (Mtd); 8, methylene-H_4_MPT reductase (Mer); 9, methyl-H_4_MPT:CoM methyltransferase (Mtr); 10, methyl-CoM reductase (Mcr). Abbreviations: CoB, coenzyme B; CoM, coenzyme M; F_420_, coenzyme F_420_; Fd, ferredoxin; H_4_MPT, tetrahydromethanopterin; MF, methanofuran.

Fig 6 shows a possible biochemical pathway for the methanogenic utilization of 2-propanol with formate as an intermediate, which was reconstructed based on the gene content of *Methanospirillum* strains growing on this substrate. In the proposed metabolic pathway, energy is conserved by the membrane-bound methyl-H_4_MPT:CoM methyltransferase complex (Fig 6, reaction 9), which generates a sodium gradient that in turn drives a sodium-translocating ATP synthase [100].

Besides genes for the cytoplasmic F_420_-coupled hydrogenase (Frh, KHC33_15955–15970), genes for several membrane-associated hydrogenases were detected that probably play a role in the reduction of ferredoxin with hydrogen. The energy-conserving hydrogenase A (Eha, KHC33_14950–15015) is flanked by *fmd* genes and probably plays a role in the anaplerotic production of reduced ferredoxin for the reductive CO_2_ fixation by formylmethanofuran dehydrogenase if the availability of the CoM-S-S-CoB heterodisulfide limits methanogenesis [101]. The ion-translocating EchA-F hydrogenase (KHC33_05645–05670), on the other hand, could provide reduced ferredoxin for anabolic reactions such as gluconeogenesis. In the absence of external sources, H_2_ for the energy-dependent reduction of ferredoxin could be generated from formate by the activity of formate lyase or from F_420_H_2_ by a reversible reaction of the F_420_-coupled hydrogenase.

## Conclusions

Based on the data presented in this study the isolate J.3.6.1-F.2.7.3^T^ can be assigned to a novel species within the genus *Methanospirillum*. Distinguishing characteristics that allow a differentiation from related species are listed in Table 3. The erroneous affiliation of the *Methanospirillum* strains GP1 and SK to *M*. *hungatei* has led to some confusion about the correct identification of *M*. *hungatei*. Important attributes like the requirement for acetate or fixation of N_2_ were incorrectly assigned to *M*. *hungatei* based on results obtained with strains GP1 and SK. Therefore, the protologue of *M*. *hungatei* has also to be emended. Another difference to *M*. *hungatei* seems to be an increased sensitivity to high amounts of nitrogenous compounds, which could be reflected in a niche separation of both species.

**Table 3:** Distinguishing features of *M*. *purgamenti* sp. nov. compared to the most closely related species.

Characteristic	*M*. *purgamenti*	*M*. *hungatei*	*M*. *stamsii*	*M*. *lacunae*
Type strain	J.3.6.1-F.2.7.3^T^	JF-1^T^	Pt1^T^	Ki8-1^T^
Available isolates	3	1	1	1
Isolation source	Freshwater sediment, wastewater	Sewage sludge	Low temperature bioreactor	Puddly soil
Cell size	0.4–0.5 μm x 7–20 μm	0.4–0.5 x 7–10 μm	0.4–0.5 μm x 7–25 μm	0.5–0.6 μm x 8–26 μm
Temperature range (optimum)	20–45°C (35–37°C)	25–50°C (40–45°C)	5–37°C (20–30°C)	15–37°C (30)
pH range (optimum)	5.5–7.5 (7.0–7.2)	6.5–10.0 (6.6–7.4)	6.0–10.0 (7.0–7.5)	6.0–9.5 (7.5)
NaCl range (optimum)	0–15 g/l (3 g/l)	0–10 g/l (0 g/l)	0–17 g/l (0 g/l)	0–10 g/l (0 g/l)
Minimal doubling time	10.0–13.0 h	20.7 h	39.8 h	32.3 h
Fixation of N_2_	+^a^	N.D.	N.D.	N.D.
Complex nutrients stimulate growth	-	+	+	N.D.
Requirement for acetate	+	-	-	+
Requirement for vitamins	-	-	N.D.	N.D.
Utilization of 2-propanol	+	-	-	+^b^/-
F_420_-dependent secondary alcohol dehydrogenase gene (*adf*)	+	-	-	+
Nitrogenase genes (*nifHDK*)	+	-	+	+
G + C content	42.1–42.2 mol%	45.1 mol%	42.3 mol%	43.1 mol%
Genome size	3.39–3.52 Mb-	3.54 Mb	3.74 Mb	3.74 Mb
References	This study, [44, 70]	[44, 61, 73, 102]	[71]	[61]

Common characteristics are: Gram-negative, slender curved rods with blunt ends in wavy filaments, tubular protein sheath containing several cells, endospores are absent, polar tufts of archaella, membrane lipids are dominated by acyclic glycerol dialkyl glycerol tetraethers (GDGT-0) linked to a disaccharide unit, methanophenazine not detected, electron acceptor is CO_2_, and suitable electron donors are H_2_ and formate. Abbreviations: N.D., not determined; Mb, mega-base-pairs.

^a^ Verified with strain GP1 by laboratory tests [88].

^b^ The result was obtained with DSM 22751^T^ (this study). In the original description, the use of 2-propanol was reported as negative.

Both cultivation-independent data and the number of isolates obtained independently from different habitats indicate that *M*. *purgamenti* sp. nov. plays a more important role in the environment than *M*. *hungatei*. It is therefore advisable to use strains of this species in preference to the type strain of *M*. *hungatei* for experiments on the syntrophic degradation of organic compounds.

### Description of *Methanospirillum purgamenti* sp. nov.

*Methanospirillum purgamenti* (pur.ga.men’ti. L. gen. neut. n. *purgamenti*, of sewage, pertaining to the isolation from wastewater).

Free-living, Gram-negative, non-spore-forming, curved rod-shaped cells with blunt ends that are motile by a polar tuft of archaella. Most cells have a diameter of 0.4–0.5 μm and a length ranging from 7 to 20 μm. Several cells are surrounded by a protein sheath with striated surface. Membrane lipids contain large amounts of acyclic dibiphytanyl diglycerol tetraether, which is esterified with glycerophosphoric acid at one end and glycosidically linked to a disaccharide unit at the other end. Methanophenazine not detected. Growth occurs at temperatures between 20 and 45°C, a pH range of 5.5–7.5 and a salinity range of 0–15 g/l NaCl. Optimal conditions for growth are 35–37°C, pH 7.0–7.2 and 3 g/l NaCl. The generation time at 37°C is 10.0–13.0 h. Molecular nitrogen (N_2_) can be used as a source of nitrogen. Vitamins are not required for growth and yeast extract or peptones do not stimulate growth. High concentrations of organic nitrogen compounds inhibit growth. Strictly anaerobic metabolism with CO_2_ as electron acceptor, which is reduced to methane. Acetate is required as a carbon source. H_2_, formate and 2-propanol can be used as electron donors for methanogenesis. Trimethylammonium chloride, acetate, pyruvate, methanol, ethanol, or 1-propanol are not used as substrates with or without acetate as carbon source. Genomes of strains of this species are characterized by a size of 3.39–3.52 Mb and a DNA G+C content of 42 mol%. The type strain is J.3.6.1-F.2.7.3^T^ (= DSM 107957^T^ = NBRC 114537^T^), isolated from an anaerobic enrichment culture initially inoculated with digested sewage sludge from a sewage plant in Constance, Germany. The GenBank/EMBL/DDBJ accession numbers for the 16S rRNA gene sequence and the complete genome sequence of the type strain are PP213394 and CP075546, respectively.

### Emended description of *Methanospirillum hungatei*

The description is as given by Ferry et al. 1974 [102] and Iino et al. 2010 [61] with the following emendations. Facultative autotroph. Acetate is not required for growth. Molecular nitrogen is not fixed. Yeast extract and peptones stimulate growth. Membrane lipids contain large amounts of acyclic dibiphytanyl diglycerol tetraether, which is esterified with glycerophosphoric acid at one end and glycosidically linked to a disaccharide unit at the other end. Cytochromes and methanophenazine are not detected. The G+C content of the genomic DNA is 45 mol%. The GenBank/EMBL/DDBJ accession number for the complete genome sequence of the type strain JF-1^T^ is CP000254.

## Supporting information

S1 Fig Phylogenetic trees of the order *Methanomicrobiales* showing the placement of the isolate J.3.6.1.-F.2.7.3^T^ within the *Methanospirillaceae*. Tree topologies were reconstructed under the maximum-likelihood criterion either based on deduced amino acid sequences of the methyl-coenzyme M reductase subunit alpha (McrA) or 16S rRNA gene sequences. The respective sequences of *Methanocella paludicola* were used as outgroup (not shown). The names of the representative strains of each species are listed in S1 Table. Accession numbers are given in parenthesis. The model LG+I+G4 of protein evolution was used for reconstruction of the McrA trees using the IQ-TREE web server, while the GTRGAMMA model was applied for reconstruction of 16S rRNA gene trees using RaXML implemented in the ARB software. Bootstrap analysis was stopped after 1000 iterations and support values above 80% are shown at the respective nodes of the best-scoring trees. Background shading with different colors is used to delineate clades at the family level, as explained in Fig 1. The scale bar indicates the expected number of substitutions per site.
(PDF)

S2 Fig Synteny plot of bidirectional best hits between the genomes of the isolate J.3.6.1.-F.2.7.3^T^, *M*. *hungatei* SK, *M*. *hungatei* GP1, and *M*. *hungatei* JF-1^T^. (PDF)

S1 Table Accession numbers of sequences used to reconstruct the phylogenetic relationship of the novel strain J.3.6.1-F.2.7.3^T^ with other representatives of the order *Methanomicrobiales*. Type strains are marked with a superscript T. Note that sets of identical McrA sequences were found in *Methanoculleus marisnigri* and *Methanoculleus submarinus* (WP_011843221 and WP_011843456).
(PDF)

S2 Table Genome statistics of the isolate J.3.6.1-F.2.7.3^T^ and related strains. Numbers are based on the NCBI Prokaryotic Genome Annotation Pipeline (PGAP).
(PDF)

S3 Table Origin of 16S rRNA gene sequences available in public nucleotide databases that are nearly identical to the sequence of strain J.3.6.1-F.2.7.3^T^. (PDF)
