# Supplementary figures and images for: Description and genome analysis of a novel archaeon isolated from a syntrophic pyrite-forming enrichment culture and reclassification of Methanospirillum hungatei strains GP1 and SK as Methanospirillum purgamenti sp. nov
Source: PLoS One. 2024 Aug 26;19(8):e0308405. doi: 10.1371/journal.pone.0308405 (PMC11346949; doi:10.1371/journal.pone.0308405)

McrA

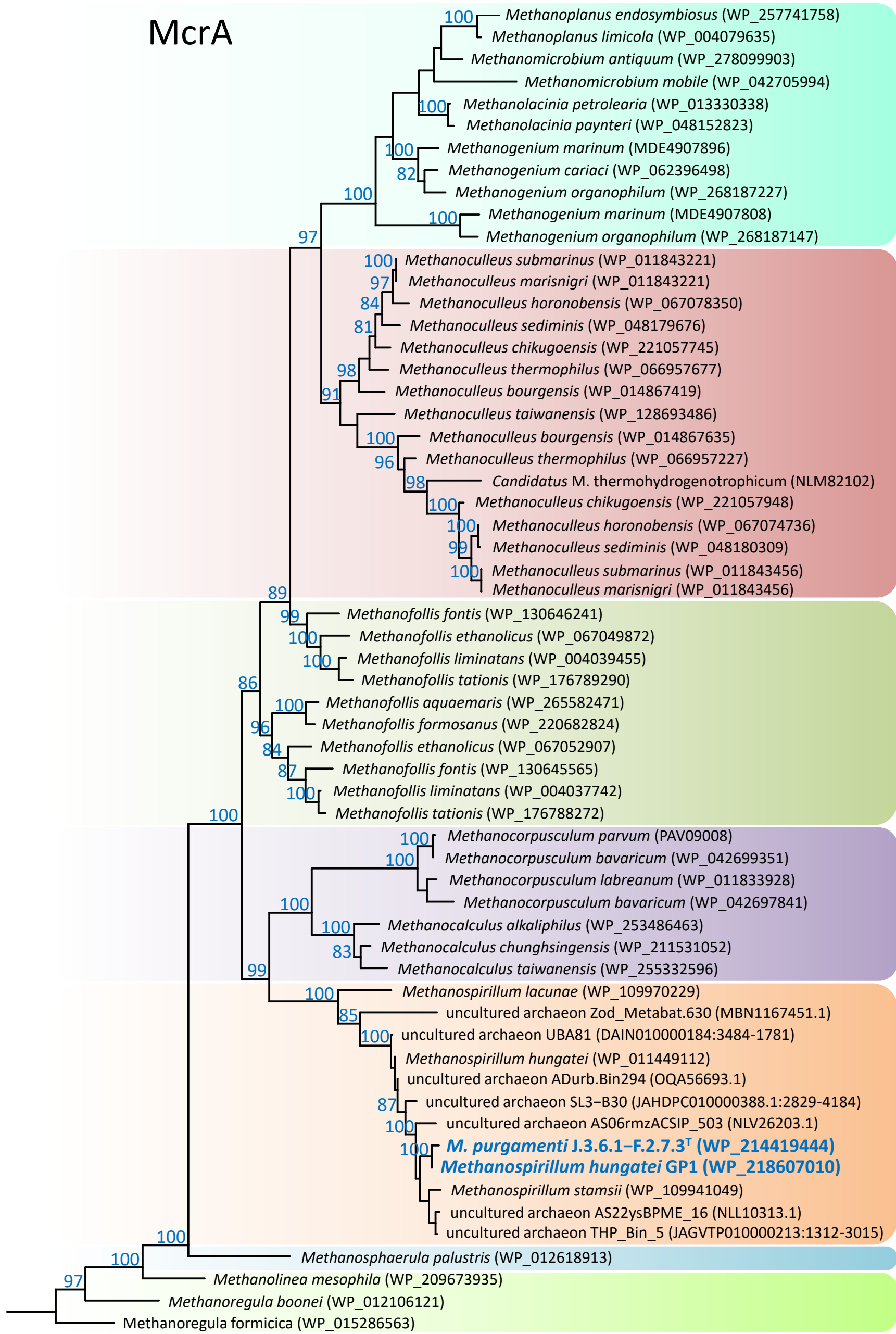

0.10

16S rRNA

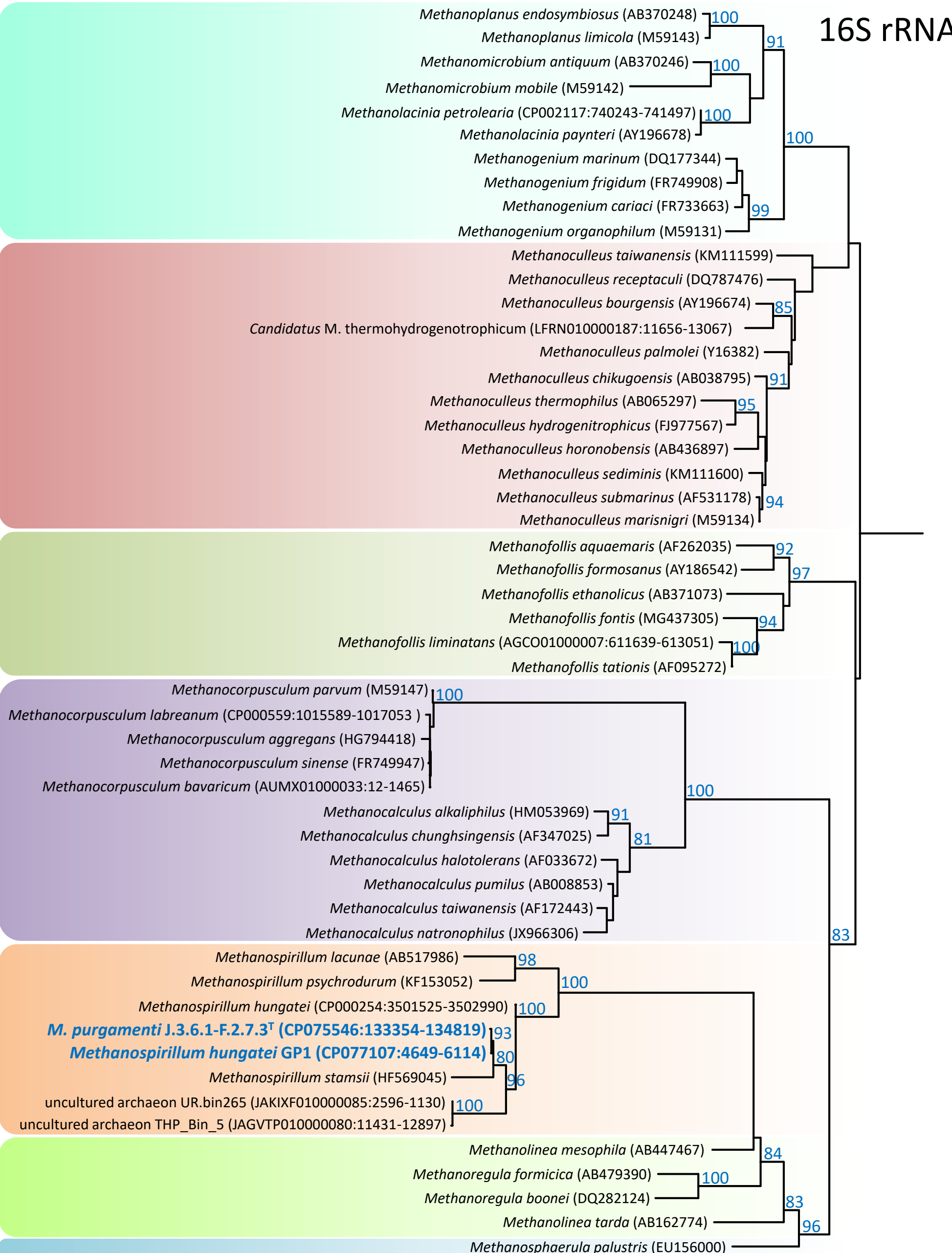

0.10

Supplement: S1 Fig — Tree topologies were reconstructed under the maximum-likelihood criterion either based on deduced amino acid sequences of the methyl-coenzyme M reductase subunit alpha (McrA) or 16S rRNA gene sequences. The respective sequences of Methanocella paludicola were used as outgroup (not shown). The names of the representative strains of each species are listed in S1 Table. Accession numbers are given in parenthesis. The model LG+I+G4 of protein evolution was used for reconstruction of the McrA trees using the IQ-TREE web server, while the GTRGAMMA model was applied for reconstruction of 16S rRNA gene trees using RaXML implemented in the ARB software. Bootstrap analysis was stopped after 1000 iterations and support values above 80% are shown at the respective nodes of the best-scoring trees. Background shading with different colors is used to delineate clades at the family level, as explained in Fig 1. The scale bar indicates the expected number of substitutions per site. (PDF) [file pone.0308405.s001.pdf]

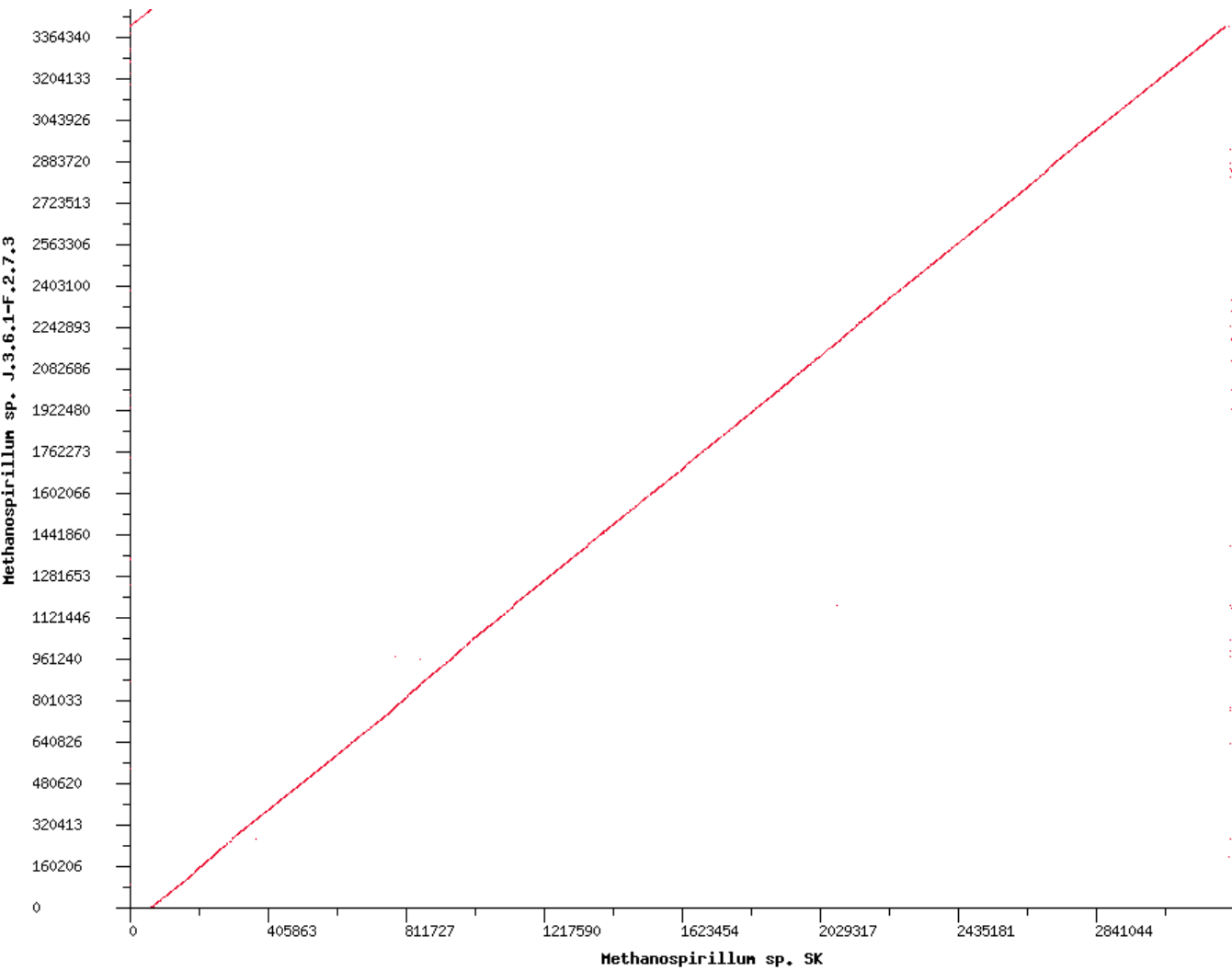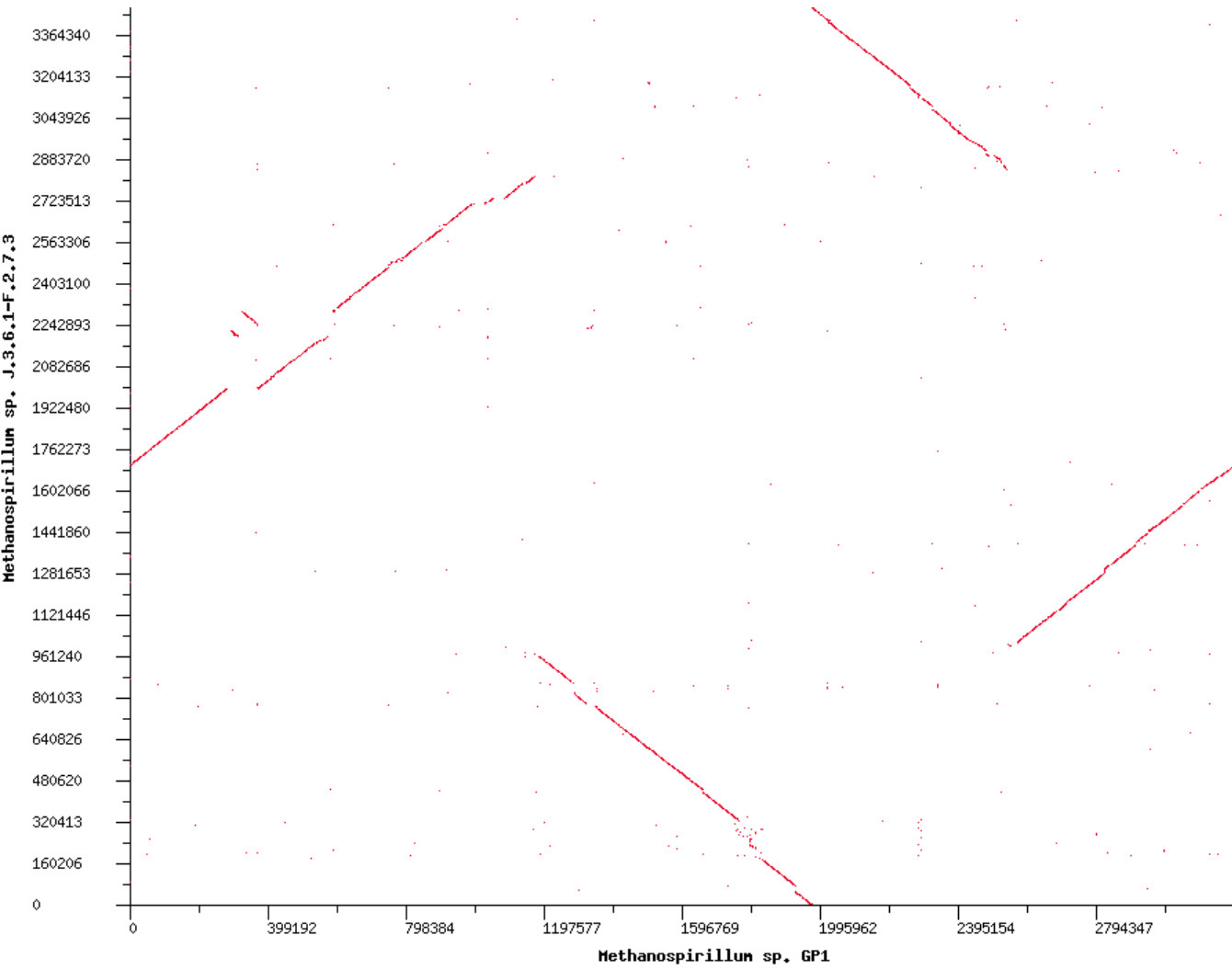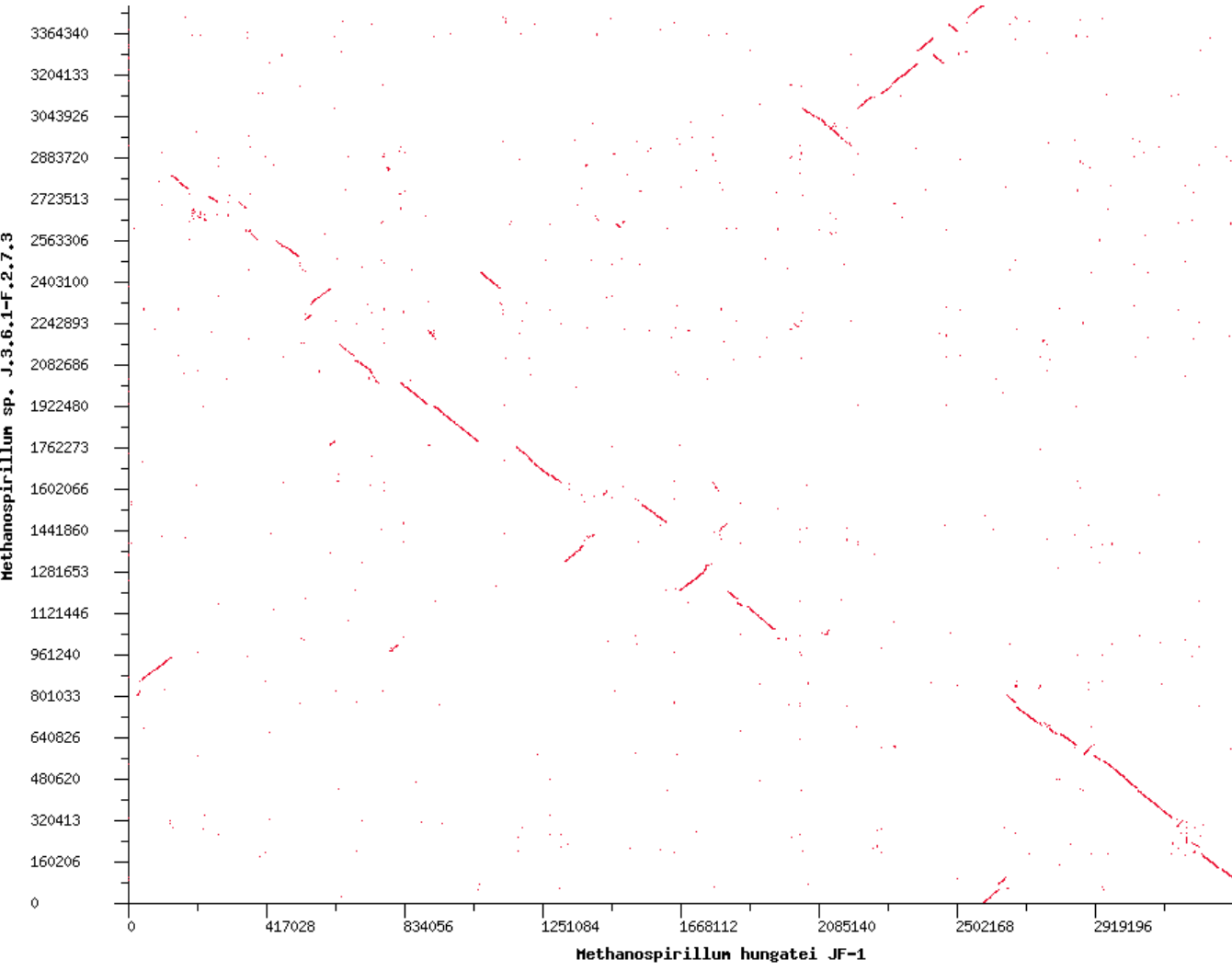

Supplement: S2 Fig — (PDF) [file pone.0308405.s002.pdf]
