# Supplementary material for: Description and genome analysis of a novel archaeon isolated from a syntrophic pyrite-forming enrichment culture and reclassification of Methanospirillum hungatei strains GP1 and SK as Methanospirillum purgamenti sp. nov
Source: PLoS One. 2024 Aug 26;19(8):e0308405. doi: 10.1371/journal.pone.0308405 (PMC11346949; doi:10.1371/journal.pone.0308405)
Supplement: S1 Table — Type strains are marked with a superscript T. Note that sets of identical McrA sequences were found in Methanoculleus marisnigri and Methanoculleus submarinus (WP_011843221 and WP_011843456). (PDF) [file pone.0308405.s003.pdf]

S1 Table. Accession numbers of sequences used to reconstruct the phylogenetic relationship of the novel isolate J.3.6.1-F.2.7.3^{T} with other representatives of the order *Methanomicrobiales*. Type strains are marked with a superscript T. Note that sets of identical McrA amino acid sequences were found in *Methanoculleus marisnigri* and *Methanoculleus submarinus* (WP_011843221 and WP_011843456).

Strain	McrA protein (> 450 aa)	16S rRNA gene (> 1200 nt)	Genome sequence	Genome assembly
**uncultured archaeon ADurb.Bin294**	OQA56693	none	MWBM00000000	GCA_002069575.1
***Methanocalculus alkaliphilus* AMF-2^{T}**	WP_253486463	HM053969	JALJYG000000000	GCF_024170505.1
***Methanocalculus chunghsingensis* K1F9705b^{T}**	WP_211531052	AF347025	JWHL00000000	GCF_018132105.1
***Methanocalculus halotolerans* SEBR 4845^{T}**	none	AF033672	none	none
***Methanocalculus natronophilus* Z-7105^{T}**	none	JX966306	none	none
***Methanocalculus pumilus* MHT-1^{T}**	none	AB008853	none	none
***Methanocalculus taiwanensis* P2F9704a^{T}**	WP_255332596	AF172443	VOTZ00000000	GCF_024372745.1
***Methanocorpusculum aggregans* MSt^{T}**	none	HG794418	none	none
***Methanocorpusculum bavaricum* DSM 4179^{T}**	WP_042699351, WP_042697841	AUMX01000033: 12-1465	AUMX00000000	GCF_000430905.1
***Methanocorpusculum labreanum* Z^{T}**	WP_011833928	CP000559: 1015589-1017053	CP000559	GCF_000015765.1
***Methanocorpusculum parvum* XII^{T}**	PAV09008	M59147	LMVO00000000	GCA_002287215.1
***Methanocorpusculum sinense* China Z^{T}**	none	FR749947	none	none
***Methanoculleus bourgensis* MS2^{T}**	WP_014867635, WP_014867419	AY196674	HE964772	GCF_000304355.2
***Methanoculleus chikugoensis* MG62^{T}**	WP_221057745, WP_221057948	AB038795	AP019781	GCF_019669965.1
***Methanoculleus horonobensis* JCM 15517^{T}**	WP_067078350, WP_067074736	AB436897	BCNY00000000	GCF_001602375.1
***Methanoculleus hydrogenitrophicus* HC^{T}**	none	FJ977567	none	none
***Methanoculleus marisnigri* JR1^{T}**	WP_011843221(A), WP_011843456(A)	M59134	CP000562	GCF_000015825.1
***Methanoculleus palmolei* INSLUZ^{T}**	none	Y16382	none	none
***Methanoculleus receptaculi* ZC-2^{T}**	none	DQ787476	none	none
***Methanoculleus sediminis* S3Fa^{T}**	WP_048179676, WP_048180309	KM111600	JXOJ00000000	GCF_001017125.1
***Methanoculleus submarinus* DSM 15122^{T}**	WP_011843221(B), WP_011843456(B)	AF531178	CP109831	GCA_025914035.1
***Methanoculleus taiwanensis* CYW4^{T}**	WP_128693486	KM111599	LHQS00000000	GCF_004102725.1
***Candidatus* Methanoculleus thermohydrogenotrophicum DTU006**	NLM82102	LFRN01000187: 11656-13067	LFRN00000000	GCA_001512375.1
***Methanoculleus thermophilus* DSM 2373^{T}**	WP_066957227, WP_066957677	AB065297	FNFT00000000	GCF_900101055.1
***Methanofollis aquaemaris* N2F9704^{T}**	WP_265582471	AF262035	CP036172	GCA_017357525.1
***Methanofollis ethanolicus* JCM 15103^{T}**	WP_067052907, WP_067049872	AB371073	BCNW00000000	GCF_001571385.1
***Methanofollis fontis* FWC-SCC2^{T}**	WP_130645565, WP_130646241	MG437305	PGCL00000000	GCF_004297185.1
***Methanofollis formosanus* ML15^{T}**	WP_220682824	AY186542	CP037968	GCF_019633745.1
***Methanofollis liminatans* DSM 4140^{T}**	WP_004039455, WP_004037742	AGCO01000007: 611639-613051	AGCO00000000	GCF_000275865.1
***Methanofollis tationis* DSM 2702^{T}**	WP_176789290, WP_176788272	AF095272	JABXWR000000000	GCF_013377755.1
***Methanogenium cariaci* JCM 10550^{T}**	WP_062396498	FR733663	BBBG00000000	GCF_001315945.1
***Methanogenium frigidum* DSM 16458^{T}**	none	FR749908	none	none
***Methanogenium marinum* DSM 15558^{T}**	MDE4907896, MDE4907808	DQ177344	JAKELO000000000	GCA_028912405.1
***Methanogenium organophilum* DSM 3596^{T}**	WP_268187227, WP_268187147	M59131	CP113361	GCA_026684035.1
***Methanolacinia paynteri* DSM 2545^{T}**	WP_048152823	AY196678	AXDV00000000	GCF_000784355.1
***Methanolacinia petrolearia* DSM 11571^{T}**	WP_013330338	CP002117: 740243-741497	CP002117	GCF_000147875.1
***Methanolinea mesophila* DSM 23604^{T}**	WP_209673935	AB447467	JAGGKR000000000	GCF_017873855.1
***Methanolinea tarda* NOBI-1^{T}**	none	AB162774	none	none
**uncultured archaeon AS22ysBPME_16**	NLL10313	none	JAAYPD000000000	GCA_012520015.1
**uncultured archaeon AS06rmzACSIP_503**	NLV26203	none	JAAYCY000000000	GCA_012729535.1
**uncultured archaeon THP_Bin_5**	JAGVTP010000213: 1312-3015	JAGVTP010000080: 11431-12897	JAGVTP000000000	GCA_019136755.1
**uncultured archaeon UBA81**	DAIN010000184: 3484-1781	none	DAIN00000000	GCA_002496135.1
***Methanomicrobium antiquum* DSM 21220^{T}**	WP_278099903	AB370246	CP091092	GCA_029633915.1
***Methanomicrobium mobile* BP^{T}**	WP_042705994	M59142	JOMF00000000	GCF_000711215.1
***Methanoplanus endosymbiosus* DSM 3599^{T}**	WP_257741758	AB370248	CP096115	GCF_024662215.1
***Methanoplanus limicola* DSM 2279^{T}**	WP_004079635	M59143	CM001436	GCF_000243255.1
***Methanoregula boonei* 6A8^{T}**	WP_012106121	DQ282124	CP000780	GCF_000017625.1
***Methanoregula formicica* SMSP^{T}**	WP_015286563	AB479390	CP003167	GCF_000327485.1
***Methanosphaerula palustris* E1-9c^{T}**	WP_012618913	EU156000	CP001338	GCF_000021965.1
**uncultured archaeon Zod_Metabat.630**	MBN1167451	none	JAFGOM000000000	GCA_016926505.1
***Methanospirillum hungatei* JF-1^{T}**	WP_011449112	CP000254: 3501525-3502990	CP000254	GCF_000013445.1
***Methanospirillum hungatei* GP1**	WP_218607010	CP077107: 4649-6114	CP077107	GCF_019263745.1
***Methanospirillum lacunae* Ki8-1^{T}**	WP_109970229	AB517986	QGMY00000000	GCF_003173355.1
**uncultured archaeon SL3-B30**	JAHDPC010000388.1: 2829-4184 (partial)	none	JAHDPC000000000	GCA_018434535.1
**uncultured archaeon UR.bin265**	none	JAKIXF010000085: 2596-1130	JAKIXF000000000	GCA_022709205.1
***Methanospirillum purgamenti* J.3.6.1-F.2.7.3^{T}**	WP_214419444	CP075546: 133354-134819	CP075546	GCF_018502485.1
***Methanospirillum psychrodurum* X-18^{T}**	none	KF153052	none	none
***Methanospirillum stamsii* Pt1^{T}**	WP_109941049	HF569045	QGMZ00000000	GCF_003173335.1
***Methanocella paludicola* SANAE^{T} (outgroup)**	WP_012899268	AB196288	AP011532	GCF_000011005.1
