# Supplementary material for: Description and genome analysis of a novel archaeon isolated from a syntrophic pyrite-forming enrichment culture and reclassification of Methanospirillum hungatei strains GP1 and SK as Methanospirillum purgamenti sp. nov
Source: PLoS One. 2024 Aug 26;19(8):e0308405. doi: 10.1371/journal.pone.0308405 (PMC11346949; doi:10.1371/journal.pone.0308405)
Supplement: S2 Table — Numbers are based on the NCBI Prokaryotic Genome Annotation Pipeline (PGAP). (PDF) [file pone.0308405.s004.pdf]

S2 Table. Genome statistics of the isolate J.3.6.1-F.2.7.3^T and related strains, including the type strains *M. hungatei* JF-1^T, *M. stamsii* Pt1^T, and *M. lacunae* Ki8-1^T. Numbers are based on the NCBI Prokaryotic Genome Annotation Pipeline (PGAP).

**Attribute**	**J.3.6.1-F.2.7.3^T**	**SK**	**GP1**	**JF-1^T**	**Pt1^T**	**Ki8-1^T**
**Accession No.**	CP075546	JAXCMI000000000	CP077107	CP000254	QGMZ00000000	QGMY00000000
**Genome size (bp)**	3,524,547	3,449,840	3,393,136	3,544,738	3,740,742	3,743,701
**G + C content (mol%)**	42.1	42.1	42.2	45.1	42.3	43.1
**Contigs**	1	72	1	1	89	25
**N50 (bp)**	-	124,523	-	-	113,561	451,884
**L50**	-	9	-	-	11	3
**Genome coverage**	181.2x	100x	170.0x	14.5x	42.0x	150.0x
**Total genes**	3,395	3,324	3,266	3,415	3,635	3,467
**Protein-coding genes**	3,271	3,254	3,139	3,317	3,520	3,377
**Genes assigned to COGs** [a]	2,535	2,488	2,491	1,872	2,695	2,645
**RNA genes**	71	56	70	67	58	53
**rRNAs (5S, 16S, 23S)**	6, 4, 4	3, 1, 1	5, 4, 4	6, 4, 4	5, 1, 1	1, 1, 1
**CRISPR arrays**	3	4	2	6	2	0

[a] Based on data provided by the JGI Integrated Microbial Genomes (IMG) database.
