# Supplementary material for: Description and genome analysis of a novel archaeon isolated from a syntrophic pyrite-forming enrichment culture and reclassification of Methanospirillum hungatei strains GP1 and SK as Methanospirillum purgamenti sp. nov
Source: PLoS One. 2024 Aug 26;19(8):e0308405. doi: 10.1371/journal.pone.0308405 (PMC11346949; doi:10.1371/journal.pone.0308405)
Supplement: S3 Table — (PDF) [file pone.0308405.s005.pdf]

**S3 Table.** Origin of isolates or clones that are closely related to the isolate J.3.6.1-F.2.7.3$^T$ based on the 16S rRNA sequence identity.

| Subject Acc. No. | Percent Identity | Alignment length | Mismatches | Source | Isolate or Clone |
|---|---|---|---|---|---|
| **JAXCMI000000000** | 100.00 | 1466 | 0 | Freshwater mud | *M. hungatei* strain SK |
| **HF955499** | 100.00 | 1321 | 0 | Anaerobic LCFA-degrading enrichment culture | A1-lcfa |
| **CP077107** | 99.93 | 1466 | 1 | Pear waste fermenter | *M. hungatei* strain GP1 |
| **AB232796** | 99.93 | 1394 | 1 | Propionate-degrading methanogenic consortium | PMA05 |
| **AB236085** | 99.92 | 1320 | 1 | Anaerobic granular sludge | MP-H2-B |
| **AB236073** | 99.92 | 1319 | 1 | River sediment | AR-Eth-B |
| **AB244743** | 99.86 | 1394 | 2 | Anaerobic glycerol-feed reactor | GLY2A01 |
| **AB248618** | 99.79 | 1394 | 3 | Anaerobic butyrate degrading reactor | BHA08 |
| **AB232798** | 99.78 | 1394 | 3 | Propionate-degrading methanogenic consortium | PHA01 |
| **MH712280** | 99.78 | 1343 | 3 | Anaerobic digester fed with agro-industrial waste | *Methanospirillum* sp. strain $T_5$3BJ |
| **AB092917** | 99.71 | 1395 | 4 | Propionate-degrading methanogenic consortium | BA03 |
| **JN030684** | 99.65 | 1434 | 5 | Indian continental shelf | sagar106 |
| **JN030692** | 99.65 | 1422 | 5 | Indian continental shelf | sagar114 |
| **JN030620** | 99.64 | 1389 | 5 | Indian continental shelf | sagar42 |
| **JN030622** | 99.64 | 1382 | 5 | Indian continental shelf | sagar44 |
| **JN030621** | 99.64 | 1379 | 5 | Indian continental shelf | sagar43 |
| **JN030693** | 99.58 | 1443 | 6 | Indian continental shelf | sagar115 |
| **AB232799** | 99.57 | 1395 | 4 | Propionate-degrading methanogenic consortium | PHA02 |
| **JN030614** | 99.50 | 1390 | 7 | Indian continental shelf | sagar36 |
| **JN030612** | 99.50 | 1386 | 7 | Indian continental shelf | sagar34 |
| **JN030618** | 99.50 | 1387 | 7 | Indian continental shelf | sagar40 |
| **JN030619** | 99.42 | 1388 | 8 | Indian continental shelf | sagar41 |
| **JN030623** | 99.42 | 1377 | 8 | Indian continental shelf | sagar45 |
| **JN030617** | 99.42 | 1376 | 8 | Indian continental shelf | sagar39 |
| **JN030681** | 99.38 | 1449 | 9 | Indian continental shelf | sagar103 |
| **JN030686** | 99.37 | 1419 | 9 | Indian continental shelf | sagar108 |
| **JN030615** | 99.35 | 1385 | 9 | Indian continental shelf | sagar37 |
| **JN030613** | 99.35 | 1383 | 9 | Indian continental shelf | sagar35 |
| **JN030616** | 99.34 | 1371 | 9 | Indian continental shelf | sagar38 |
| **JN030685** | 99.29 | 1415 | 10 | Indian continental shelf | sagar107 |
| **JN030683** | 99.10 | 1443 | 13 | Indian continental shelf | sagar105 |
